# Comparative Study of the Molecular Characterization, Evolution, and Structure Modeling of Digestive Lipase Genes Reveals the Different Evolutionary Selection Between Mammals and Fishes

**DOI:** 10.3389/fgene.2022.909091

**Published:** 2022-08-04

**Authors:** Shu-Lin Tang, Xu-Fang Liang, Shan He, Ling Li, Muhammad Shoaib Alam, Jiaqi Wu

**Affiliations:** ^1^ College of Fisheries, Chinese Perch Research Center, Huazhong Agricultural University, Wuhan, China; ^2^ Engineering Research Center of Green Development for Conventional Aquatic Biological Industry in the Yangtze River Economic Belt, Ministry of Education, Wuhan, China

**Keywords:** digestive lipase gene, pancreatic lipase, bile salt-activated lipase, fish, genome, evolution

## Abstract

Vertebrates need suitable lipases to digest lipids for the requirement of energy and essential nutrients; however, the main digestive lipase genes of fishes have certain controversies. In this study, two types of digestive lipase genes (pancreatic lipase (*pl*) and bile salt-activated lipase (*bsal*)) were identified in mammals and fishes. The neighborhood genes and key active sites of the two lipase genes were conserved in mammals and fishes. Three copies of *PL* genes were found in mammals, but only one copy of the *pl* gene was found in most of the fish species, and the *pl* gene was even completely absent in some fish species (e.g., zebrafish, medaka, and common carp). Additionally, the hydrophobic amino acid residues (Ile and Leu) which are important to pancreatic lipase activity were also absent in most of the fish species. The *PL* was the main digestive lipase gene in mammals, but the *pl* gene seemed not to be the main digestive lipase gene in fish due to the absence of the *pl* gene sequence and the important amino acid residues. In contrast, the *bsal* gene existed in all fish species, even two to five copies of *bsal* genes were found in most of the fishes, but only one copy of the *BSAL* gene was found in mammals. The amino acid residues of bile salt-binding sites and the three-dimensional (3D) structure modeling of Bsal proteins were conserved in most of the fish species, so *bsal* might be the main digestive lipase gene in fish. The phylogenetic analysis also indicated that *pl* or *bsal* showed an independent evolution between mammals and fishes. Therefore, we inferred that the evolutionary selection of the main digestive lipase genes diverged into two types between mammals and fishes. These findings will provide valuable evidence for the study of lipid digestion in fish.

## Introduction

Animals must receive adequate nutrition to support their normal growth and development, lipids attract more attention due to their crucial role in metabolism/nutrition and their complex physiological process (Cai et al., 2017; Anderson et al., 2018; Cruz et al., 2020). Lipids are vital components for energy, and cell membrane structures and work as messenger molecules; they also play crucial roles in animal growth and reproduction (Wu et al., 2010; Zhu et al., 2013; González-Félix M. L. et al., 2018; Gao et al., 2019). Adequate lipids depend on efficient ingestion and digestion, which relies on various types of digestive lipases (Navarro-Guillén et al., 2015; Kulminskaya & Oberer, 2020). As the third-largest enzyme group, lipase is a key enzyme for lipids digestion (Kurtovic et al., 2009; Basheer et al., 2011; Bouchaâla et al., 2015; Cerk et al., 2018), especially for hydrolyzing triacylglycerides (TAGs), glycerophospholipids (GPs) and esters of cholesterol (Navvabi et al., 2018; Morshedi et al., 2021). Here we focused on the two types of digestive lipase (E.C. 3.1.1.3) genes, pancreatic lipase gene (*pl*) and bile salt-activated lipase gene (*bsal*), they mainly catalyze the hydrolysis of triacylglycerols into glycerol and fatty acids (Sæle et al., 2010; Holmes and Cox, 2011; Achouri et al., 2020; Qiu et al., 2020).

In mammals, the most important digestive lipase genes are the pancreatic lipase gene (*PL*) and two genes closely related to the *PL* gene, namely pancreatic lipase-related protein 1 (*PLRP1*) and pancreatic lipase-related protein 2 (*PLRP2*) (Kurtovic et al., 2009; Zhu et al., 2021). Meanwhile, the bile salt-activated lipase (*BSAL*) also have a wide range of role in the hydrolyzing of TAGs, GPs, cholesterol esters, and lipid-soluble vitamins (Holmes & Cox, 2011; Navvabi et al., 2018). In contrast, in teleost fishes, the most important digestive lipase may be the bile salt-activated lipase (*bsal*). Firstly, it has a certain controversy about the existence of a real pancreatic lipase in fishes (Rønnestad et al., 2013; Anderson et al., 2018; Yanes-Roca et al., 2018), especially the lipase purified from fishes seemed to require bile salts for activation (Tocher, 2003). Additionally, until now, no reports have confirmed whether the main digestive lipase is pancreatic lipase or bile salt-activated lipase in fish. However, the bile salt-activated lipase, also called carboxyl ester lipase (*cel*), has been suggested to be the most important digestive lipase in fish species (Murashita et al., 2014; Fuentes-Quesada & Lazo, 2018; Sæle et al., 2018; Qiu et al., 2020; Morshedi et al., 2021; Sterzelecki et al., 2021). Secondly, the absence of the *pl* gene was shown in the zebrafish genome (Sæle et al., 2018). Although, most fishes had a *plrp* gene (a copy of *pl*), according to some reports (Sæle et al., 2010; Rønnestad et al., 2013) and annotation in NCBI, these *plrp* genes might produce inactive pancreatic lipases in teleosts, these pancreatic lipases might not possess the lipolytic function due to mutations in the domain (Sæle et al., 2010; Sæle et al., 2018). Moreover, pancreatic lipase is a colipase-dependent lipase, colipase is required for lipid binding and allows the pancreatic lipase to carry out the function of hydrolyzing lipids (Smichi et al., 2017; Anderson et al., 2018; González-Félix ML. et al., 2018; Achouri et al., 2020). The absence of the colipase gene (*clps*) also can be found in zebrafish (*Danio rerio*), medaka (*Oryzias latipe*), Japanese pufferfish (*Takifugu rubripes*), and three-spined stickleback (*Gasterosteus aculeatus*) (Sæle et al., 2010; Smichi et al., 2017; Anderson et al., 2018; Sæle et al., 2018).

In the light of the above information, the main digestive lipase gene seemed to show a complete difference between mammals and fishes. In this study, we identified the two types of digestive lipase genes (*pl* and *bsal*) in several vertebrates. We predicted the amino acid sequences, structures, protein models, as well as the phylogenetic relationship for these lipase genes. These results could be helpful for the understanding of the function and evolution of digestive lipase genes in the vertebrate.

## Materials and Methods

### Databases and Data Mining

The vertebrate digestive lipase genes were collected from the following online database centers, the National Center for Biotechnology Information (NCBI) (http://blast.ncbi.nlm.nih.gov/) and/or Ensembl (http://asia.ensembl.org/index.html). The obtained data contain the information of the species including human (*Homo sapiens*), mouse (*Mus musculus*), zebrafish (*Danio rerio*), medaka (*Oryzias latipes*), common carp (*Cyprinus carpio*), Nile tilapia (*Oreochromis niloticus*), spotted gar (*Lepisosteus oculatus*), European eel (*Anguilla anguilla*), northern pike (*Esox lucius*), Atlantic cod (*Gadus morhua*), Atlantic salmon (*Salmo salar*), rainbow trout (*Oncorhynchus mykiss*), largemouth bass (*Micropterus salmoides*), Asian seabass (*Lates calcarifer*), pufferfish (*Takifugu rubripes*), Japanese flounder (*Paralichthys olivaceus*), yellow catfish (*Tachysurus fulvidraco*) and channel catfish (*Ictalurus punctatus*). The mandarin fish (*Siniperca chuatsi*) genome was sequenced using Single-Molecule Real-Time (SMRT), and the raw sequencing data of the genome was published at NCBI (PRJNA513951) (He et al., 2020). European seabass (*Dicentrarchus labrax*) sequences were obtained from the seabass genome website: http://seabass.mpipz.mpg.de/. The Basic Local Alignment Search Tool (BLAST) studies were used to recognize the similar lipase sequences (http://blast.ncbi.nlm.nih.gov/Blast.cgi), and the sequences of humans and zebrafish were used as a pattern to recognize the other vertebrate lipase gene sequences. The names, accession numbers, and sequences of all lipase genes were shown in Supplementary Table S1.

### The Approach of Pseudogene Identification

The sequences of exons were collected from the NCBI database. The open reading frames (ORFs) were predicted with the help of an online tool (https://www.ncbi.nlm.nih.gov/orffinder/), and the premature termination codon appeared in the sequences of exons in a pseudogene.

### The Confirmation of Absent Lipase Genes

The neighboring genes (*pitx3* and *hspa12a*) of *pl* gene were conserved in fishes, so the flanking sequences of *pitx3* and *hspa12a* genes were obtained from zebrafish, medaka, common carp, and Nile tilapia genomes. In order to identify whether these flanking sequences were the sequences of *pl* gene or not, the flanking sequences were aligned with other fish *pl* gene sequences, and the flanking sequences were also executed from BLAST search in the NCBI database.

### Phylogenetic Analysis

Multiple sequence alignment was carried out by ClustalW in MEGA X (Kumar et al., 2018; Tang et al., 2022). The alignment was visually inspected and manually adjusted. The phylogenetic analyses were inferred by using the Maximum Likelihood method based on a WAG + G + I model. The reliability of tree topology was repeated by the bootstrap analysis with 1000 rounds.

### Sequence and Structure Analysis

Amino acid sequences were aligned by using CLC Sequence Viewer 6 (CLC bio, Aarhus, Denmark) (Jesus et al., 2017). Protein domains were predicted with the simple modular architecture research tool (SMART) version 4.0. The presumed tertiary structures were established using the SWISS-MODEL prediction algorithm and displayed by PyMOL version 0.97.

## Results

### Digestive Lipase Gene Identification and Syntenic Analysis

**FIGURE 1:**
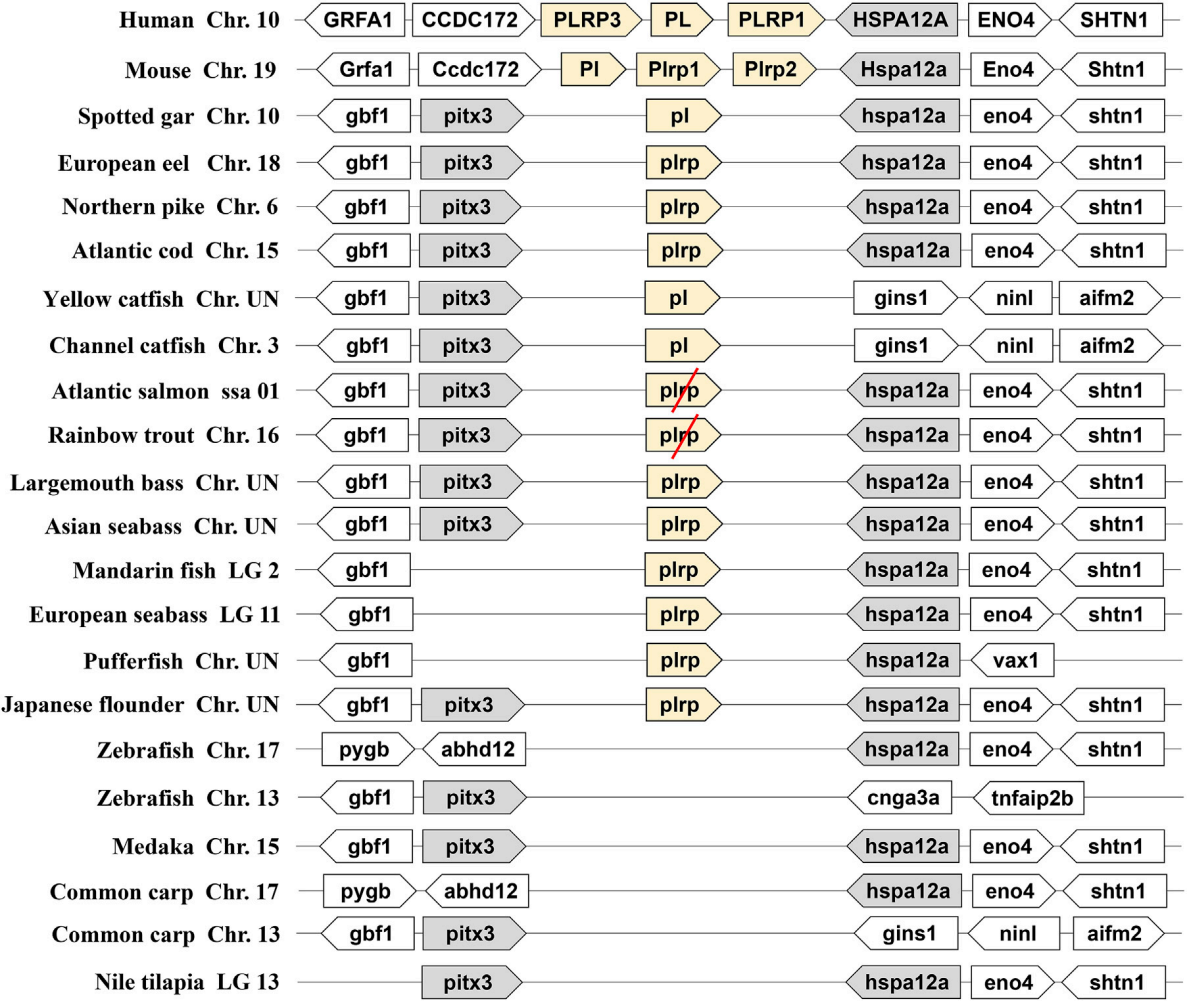
Synteny of pancreatic lipase genes (light yellow) in mammals and fishes. The conserved neighborhood genes (*pitx3* and *hspa12a*) were marked with gray. The pseudogenes were marked with a red slash.

We identified the two types of digestive lipase genes in vertebrate genomes, the results showed that the *PL* gene occurred a tandem duplication in the human and mouse genomes. They had three copies of *PL* genes (one *PL* and two *PLRP*), the three copies of *PL* genes named *PL*, *PLRP1,* and *PLRP3* in human, and these copies are located on chromosome 10. The three copies of *PL* genes are named *Pl*, *Plrp1,* and *Plrp2* in mouse, and these copies are located on chromosome 19. The flanking genes of these *PL* genes were conserved in the human and mouse (Figure 1). However, only one copy of *pl* gene was found in fishes, even the *pl* gene was absent in some fish species (zebrafish, medaka, common carp, and Nile tilapia) (Figure 1). The *pl* genes of fishes are located on different chromosomes, but the flanking genes of these *pl* genes were conserved in most of the fishes species, the neighboring genes were *gbf1*, *pitx3*, *hspa12a,* and *eno4* (Figure 1). Interestingly, although the fourth-whole-genome duplication (4-WGD) event occurred in the ancestral genome of Salmoniformes and common carp, the *pl* genes of Atlantic salmon and rainbow trout became pseudogenes, and the premature termination codon appeared in the second exon in Atlantic salmon and in the third exon in rainbow trout, respectively (Figure 1 and Supplementary Figure S1). The common carp even lost the *pl* gene (Figure 1).

**FIGURE 2:**
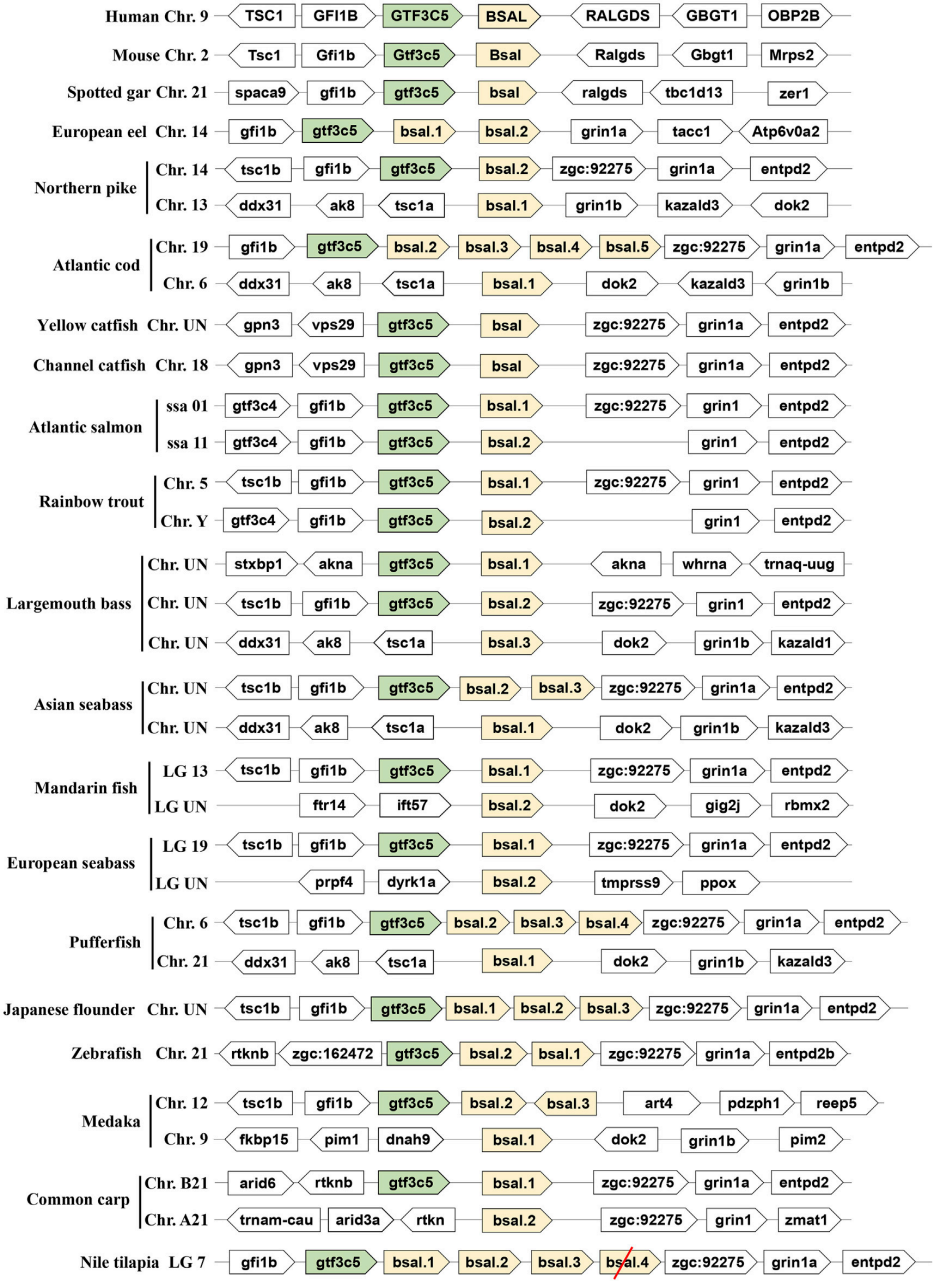
Synteny of bile salt-activated lipase genes (light yellow) in mammals and fishes. The conserved neighborhood genes (*gtf3c5*) were marked with green. The pseudogene was marked with a red slash.

As another important digestive lipase, only one copy of the *BSAL* gene existed in the human and mouse genomes, the *BSAL* genes of human and mouse are located on chromosomes 9 and 2, respectively (Figure 2). However, two to five copies of *bsal* gene existed in most fishes and these copies had a tandem duplication or one copy was translocated to different chromosomes (Figure 2). The flanking genes of *bsal* gene were conserved in most of the fish species. It was worth noting that *bsal* gene existed in all the fish species, even two to five copies appeared in most of the fishes, this situation was contrary to the *pl* gene in the fishes. Interestingly, one *pl* gene and one *bsal* gene coexisted in spotted gar, yellow catfish, and channel catfish genomes (Figures 1, 2).

### Phylogenetic Analysis of Digestive Lipase Genes

**FIGURE 3:**
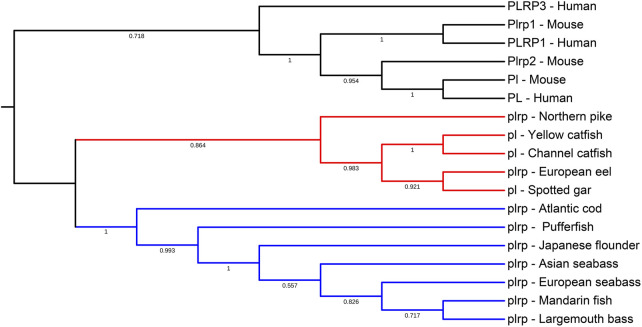
Phylogenetic relationship of pancreatic lipase genes in mammals and fishes with based on the ML method. The bootstrap test (1000 replicates) scores are shown with numbers. The blackline represents the group of *PL* genes in mammals, the red line represents the first group of *pl* genes in fishes, and the blue line represents the second group of *pl* genes in most of the fishes.

**FIGURE 4:**
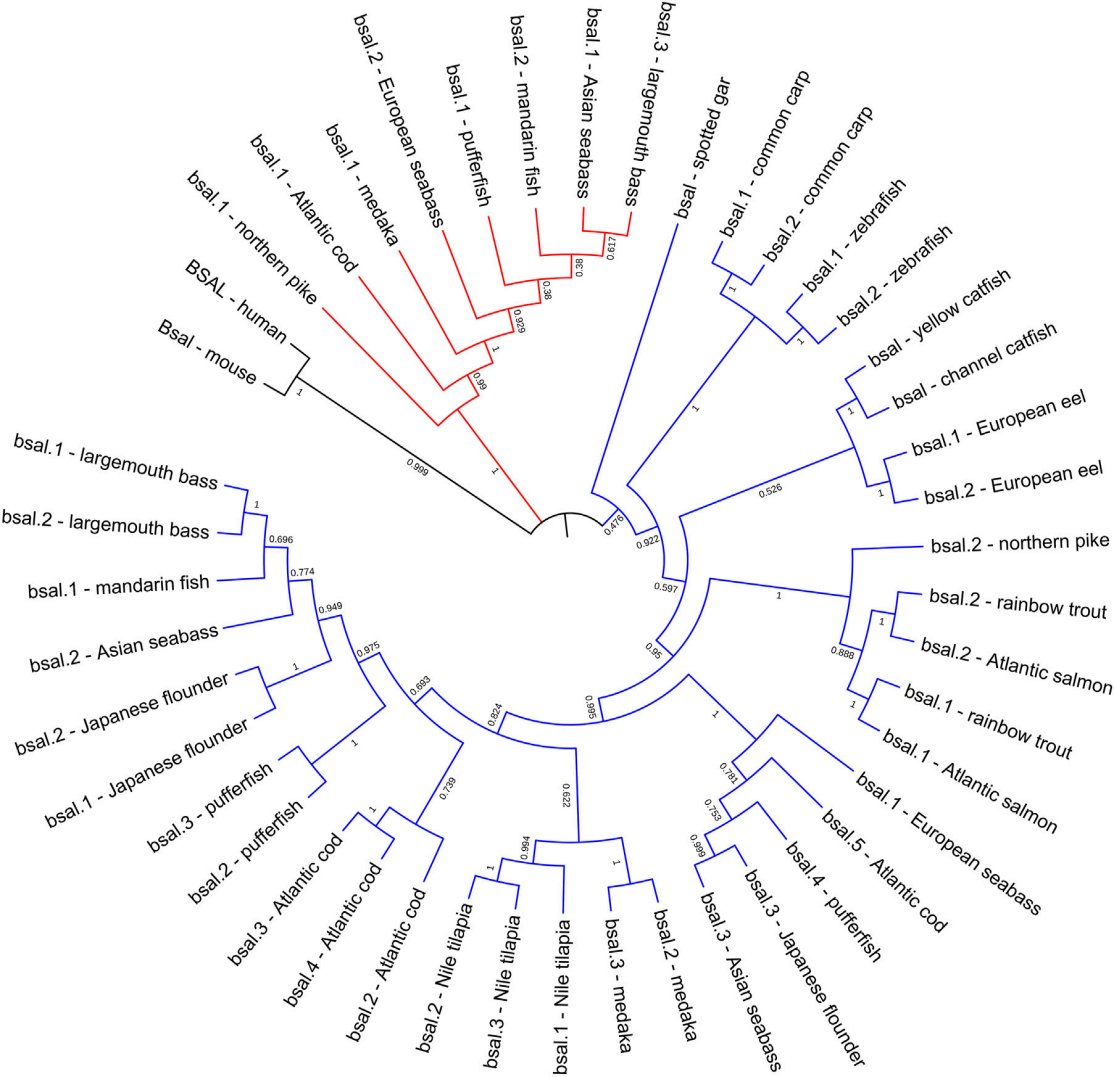
Phylogenetic relationship of bile salt-activated lipase genes in mammals and fishes with based on the ML method. The bootstrap test (1000 replicates) scores are shown with numbers. The black line represents the group of *BSAL* genes in mammals. The fish *bsal* genes whose neighborhood gene was conserved were shown with the blue line, the other *bsal* copies were shown with the red line.

To examine the evolutionary relationships among the vertebrate digestive lipase genes, we used the MEGA X program to align and construct the phylogenetic trees, the results are shown in Figures 3, 4. According to the phylogenetic analysis, pancreatic lipase genes diverged into three groups, the *PL* genes of human and mouse were grouped together as the outgroup. The *pl* genes of spotted gar, European eel, and northern pike were grouped together with catfishes, and the other fish *pl* genes (all these were *plrp* genes) were grouped together (Figure 3).

The phylogenetic tree also showed that bile salt-activated lipase genes diverged into four groups, as expected, the *BSAL* genes of human and mouse were grouped together as the outgroup. But the *bsal* genes of fishes had three groups, the *bsal* gene of spotted gar was independently evolved and also could be seen as an outgroup in fish, other fish *bsal* genes diverged into two groups (Figure 4). Combined with the results of syntenic analysis, we found that the *bsal* genes, which the neighboring gene was *gtf3c5,* were grouped together as a big group, and the *bsal* genes, which were translocated to another chromosome, were grouped together (Figures 2, 4).

### Sequence Alignment and Analysis of Digestive Lipase Genes

**FIGURE 5:**
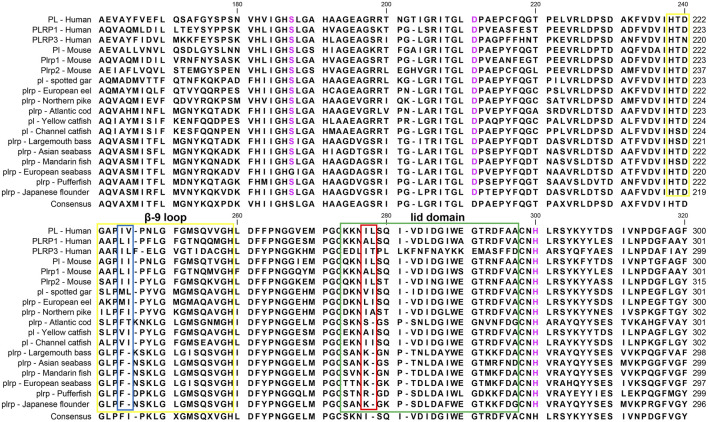
Partial amino acid sequence alignments of pancreatic lipases for humans and fishes. Boxes in green and yellow represent the lid domain and β-9 loop of pancreatic lipase, respectively. Active site triad residues Ser, Asp, and His were marked with pink. The red and blue boxes represent the absence of Ile and Leu in the lid domain and β-9 loop, respectively. The dashes (-) represent gaps found upon sequence alignment. Numbers refer to the amino acid were located at the end of each line.

**FIGURE 6:**
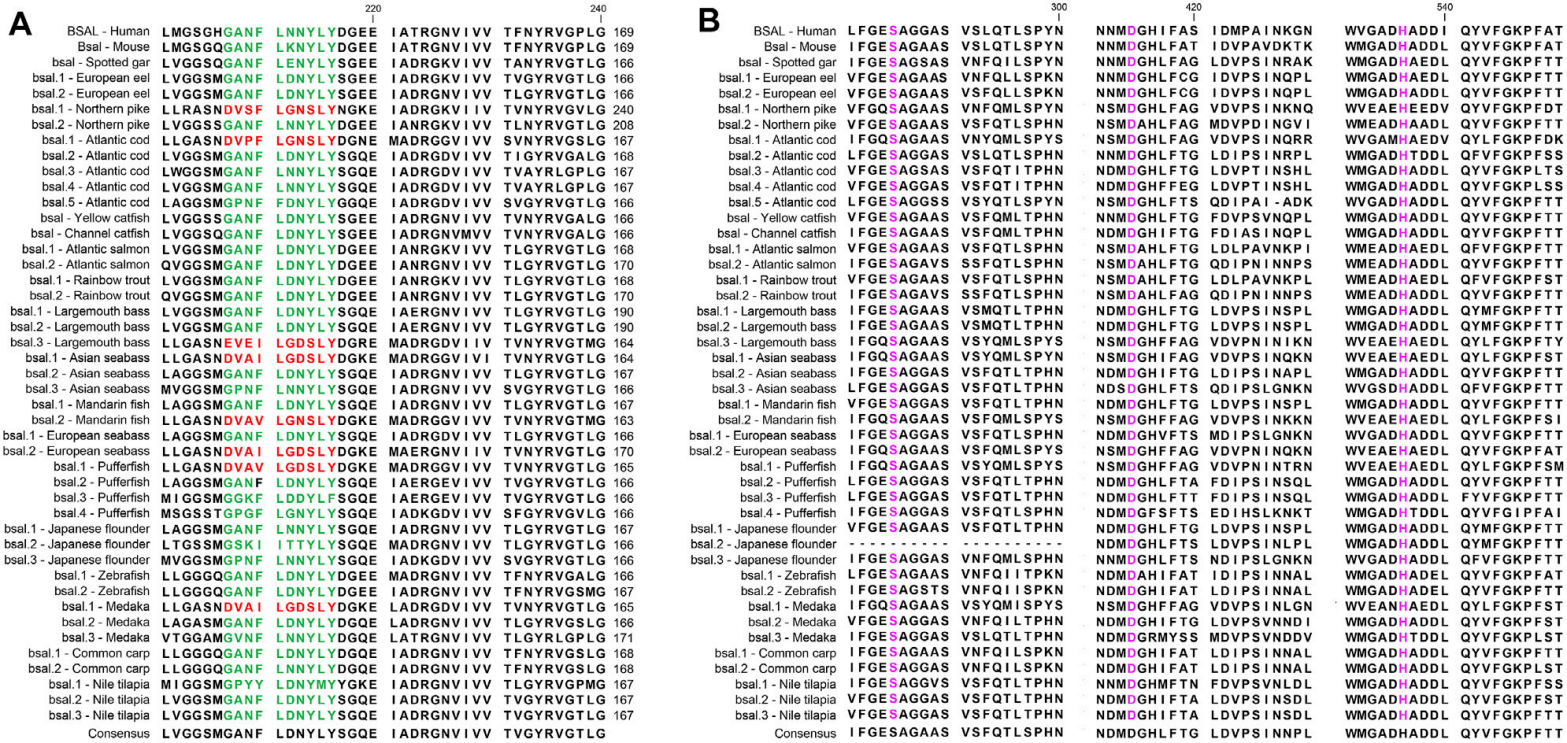
Partial Amino acid sequence alignments of bile salt-activated lipases for humans and fishes. **(A)** The correct amino acid residues of the bile salt-binding site (GANFLXNYLY) were marked with green, and other types of amino acid residues in the bile salt-binding site were marked with red. **(B)** Active site triad residues Ser, Asp, and His were marked with pink. The dashes (-) represent gaps found upon sequence alignment. Numbers refer to the amino acid were located at the end of each line.

The multiple sequence alignments of vertebrate digestive lipase genes were shown in Figures 5, 6. As the results showed, the amino acid residues of the catalytic triad (Ser-Asp-His) in pancreatic lipase were conserved in all species (Figure 5). But the lid domain, as the important interfacial activation region in pancreatic lipase, had some differences between mammals and fishes. The hydrophobic amino acid residues (Ile and Leu) were absent in some fish species (e.g., Atlantic cod, mandarin fish, seabass, pufferfish, and Japanese flounder), the Ile and Leu were replaced by Ser, Lys, or Arg (Figure 5). The same situation also could be seen in the β-9 loop, which also plays an important role in interaction with the interface, the Ile and Leu were replaced by Phe (Figure 5). Interestingly, these fishes, which lost the Ile and Leu, were grouped together in the phylogenetic analysis (Figure 3).

Several key amino acid residues of bile salt-activated lipase had been recognized (Figure 6), and the important region of the bile salt-binding site (GANFLXNYLY) was present in all species. The catalytic triad for the active sites (Ser-Asp-His) was also conserved in mammals and fishes. However, as we can see, the amino acid residues of bile salt-binding sites were different in some *bsal* copies in the fishes, the amino acid residues (GANFLXNYLY) were randomly replaced by other amino acids. Interestingly, the *bsal* genes, in which the amino acid residues changed in the bile salt-binding site, were grouped together (Figure 4), and the neighboring genes of these *bsal* gene copies were not conserved (Figure 2). The complete multiple sequence alignments of vertebrate digestive lipase genes were shown in Supplementary Figures S2, S3.

### Three-Dimensional (3D) Structure Modeling of Digestive Lipases

**FIGURE 7:**
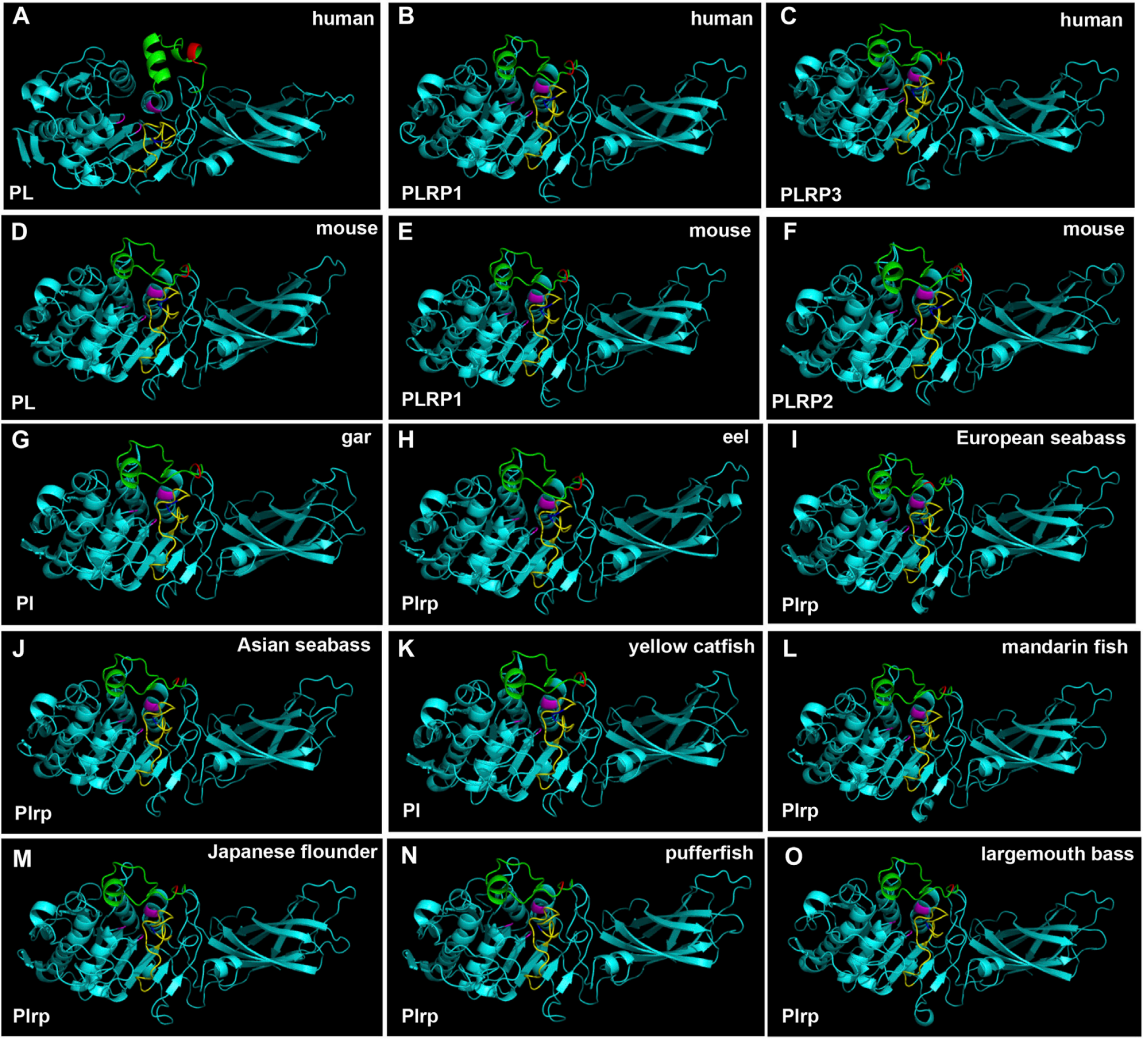
3D structures of pancreatic lipase in human, mouse, and partial fish species. The lid domain and β-9 loop of pancreatic lipase were marked with green and yellow, respectively. The regions of Ile and Leu (or replaced by other amino acids) in the lid domain and β-9 loop were marked with red and blue, respectively. Active site triad residues Ser, Asp, and His were marked with pink.

In order to obtain more information about the digestive lipase genes in fishes, the 3D structures of lipases were established by using the SWISS-MODEL prediction algorithm. The 3D structures of pancreatic lipases were conserved in mammals and fishes, except for the PL of human, other animal pancreatic lipases had very similar 3D structures (Figure 7). The catalytic triad for the active site, lid domain, and β-9 loop was present in all species, marked with pink, green and yellow, respectively (Figure 7). Although the annotations of pancreatic lipase genes were different between fishes, the 3D structures of pancreatic lipases were conserved.

**FIGURE 8:**
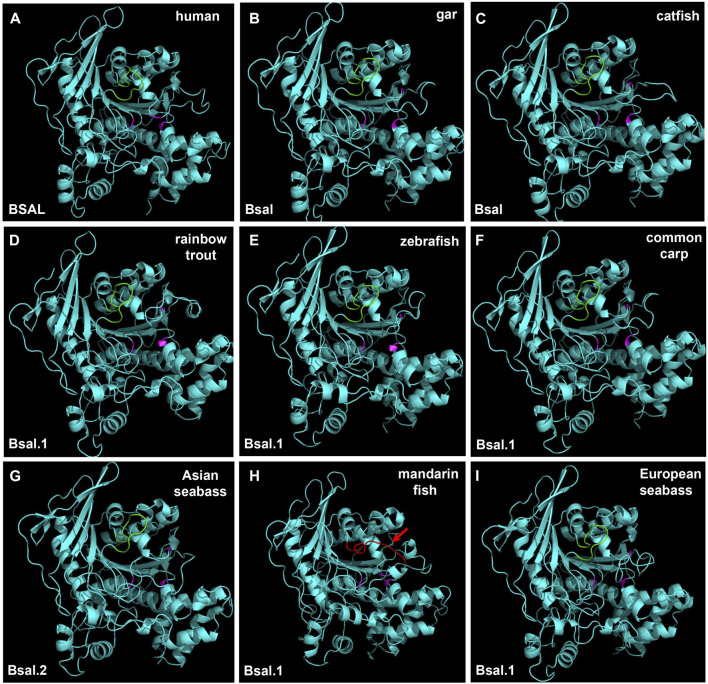
3D structures of bile salt-activated lipases in human and partial fish species. The region of amino acid residues in the bile salt-binding site (GANFLXNYLY) was marked with green. Active site triad residues Ser, Asp, and His were marked with pink. The red arrow represents the no loop structure that existed in mandarin fish **(H)**.

The region of the bile salt-binding site and catalytic triad for the active site were marked in the 3D structure of bile salt-activated lipase (Figure 8, Supplementary Figure S4). The 3D structures of bile salt-activated lipases were conserved in most of the species, but the bile salt-binding site of Bsal had a unique 3D structure in mandarin fish. Compared with other fish Bsal models, no loop structure was observed in the bile salt-binding region in mandarin fish (Figure 8H). The loop structure, which binds bile salt, might affect the lipase to digest the triacylglycerols, it might be related to the unique feeding habit of the mandarin fish.

## Discussion

Lipid is an important source of energy and a constituent of cell membranes throughout the life cycle in animals, it is considered a key nutrient during early life stages due to its high growth and development (Tocher 2003; Benedito-Palos et al., 2014; Anderson et al., 2018; Gilannejad et al., 2020). Lipases are versatile enzymes that catalyze the hydrolysis of ester linkages in lipids, such as TAGs (Karimi et al., 2010). Lipases are ubiquitous throughout animals. There are two important lipases in animals, one is pancreatic lipase (*pl*) and another is bile salt-activated lipase (*bsal*), which play an important role in hydrolyzing the ester bonds in TAGs during digestive processes (Karimi et al., 2010; Rueda-López et al., 2017). Although the reports about these two lipases are abundant in mammals, the main digestive lipase is controversial in fishes (Rønnestad et al., 2013; Anderson et al., 2018; Yanes-Roca et al., 2018). In this study, we understood the different lipid digestion genes among mammals and fishes, and this is the first report, which comprehends the digestive lipases from whole-genome in the fishes. The results showed that the pancreatic lipase gene (*pl*) just existed in part of fish species. Our findings in the present study indicated that zebrafish, medaka, common carp, and Nile tilapia lost the *pl* gene in the genome. Consistent with this result, a previous study also reported that *pl* gene was absent in zebrafish (Sæle et al., 2018). Moreover, we also found that the *pl* genes of Atlantic salmon and rainbow trout became pseudogenes. The syntenic analysis had ensured the correction of the whole genome identified in the present study, the neighboring genes were conserved in all fish species. Although most of the fish species possessed the *pl* gene, and they only had one copy of *pl* gene in the genome, the *PL* gene had occurred in gene tandem duplication and obtained three copies of the *PL* gene in mammals. The three copies of *PL* gene had been reported and confirmed in mammals (Xiao et al., 2011; Aloulou et al., 2014; Zhu et al., 2021). In contrast, as we can see, the *BSAL* gene not only existed in mammals but also existed in all fishes, even the *bsal* had occurred gene duplication in most of the fish species. Therefore, the copy number of lipase genes exhibited different models between mammals and fishes.

Meanwhile, the amino acid sequence analysis showed that the Ile and Leu were replaced by Ser, Lys, or Arg in the lid domain of pancreatic lipase in most fishes species, it also might influence the lipolytic ability of pancreatic lipase in fish (Smichi et al., 2017). However, the *bsal* gene occurred the gene duplication and obtained two to five copies in the fish species in which the Ile and Leu were absent, the phylogenetic analysis also showed that these fish species were grouped together (Figure 3). Thus, we inferred that most of the fish species had the *pl* gene, but this gene might have no or low capacity to digest the lipids, the annotation of these *pl* genes in NCBI also are inactive pancreatic lipase-related protein genes. In order to compensate for the low or inactive lipolytic ability of pancreatic lipase in these fish species, the bile salt-activated lipase gene (*bsal*) might occur the gene duplication. Some studies also believed that the *bsal* gene was the main and most important digestive lipase in Atlantic cod (*Gadus morhua*), Pacific bluefin tuna (*Thunnus orientalis*), California halibut (*Paralichthys californicus*), and yellowfin seabream (*Acanthopagrus latus*) (Sæle et al., 2010; Murashita et al., 2014; Fuentes-Quesada & Lazo, 2018; Morshedi et al., 2021). Some specific fish species, like spotted gar and catfishes, had the *pl* gene which might have the great capacity to digest the lipids, so the *bsal* did not occur gene duplication, they only had one copy of *bsal* gene. Similarly, the *BSAL* also did not occur in gene duplication in mammals. In addition, the amino acid of the lid domain in pancreatic lipase was conserved in these species, it also means that the function of pancreatic lipase might be fully-functioning (Ollis et al., 1992; Verger 1997; Smichi et al., 2017).

Therefore, we preliminarily considered the main digestive lipase gene was completely different between mammals and most fishes. We inferred that the *PL* in the ancestor of mammals possessed the lipolytic ability and obtained two other similar copies (*PLRP*), so the *BSAL* was not necessary to obtain an extra copy and retained one *BSAL* gene in mammals. However, the *pl* gene might lose the lipolytic ability in some fish ancestors, instead, the *bsal* not only played a major function in lipolytic ability in these fish species but also occurred gene duplication. Sequence alignment also revealed the amino acid residues of the bile salt-binding site were conserved in most of *bsal* genes (Figure 4). In some of the fish species, the *bsal* copies had been translocated to another chromosome, and the amino acid residues of the bile salt-binding site also changed (Figures 2, 4), it might be some mistakes happened when the *bsal* copies were translocated to another chromosome after the gene duplication occurred.

**FIGURE 9:**
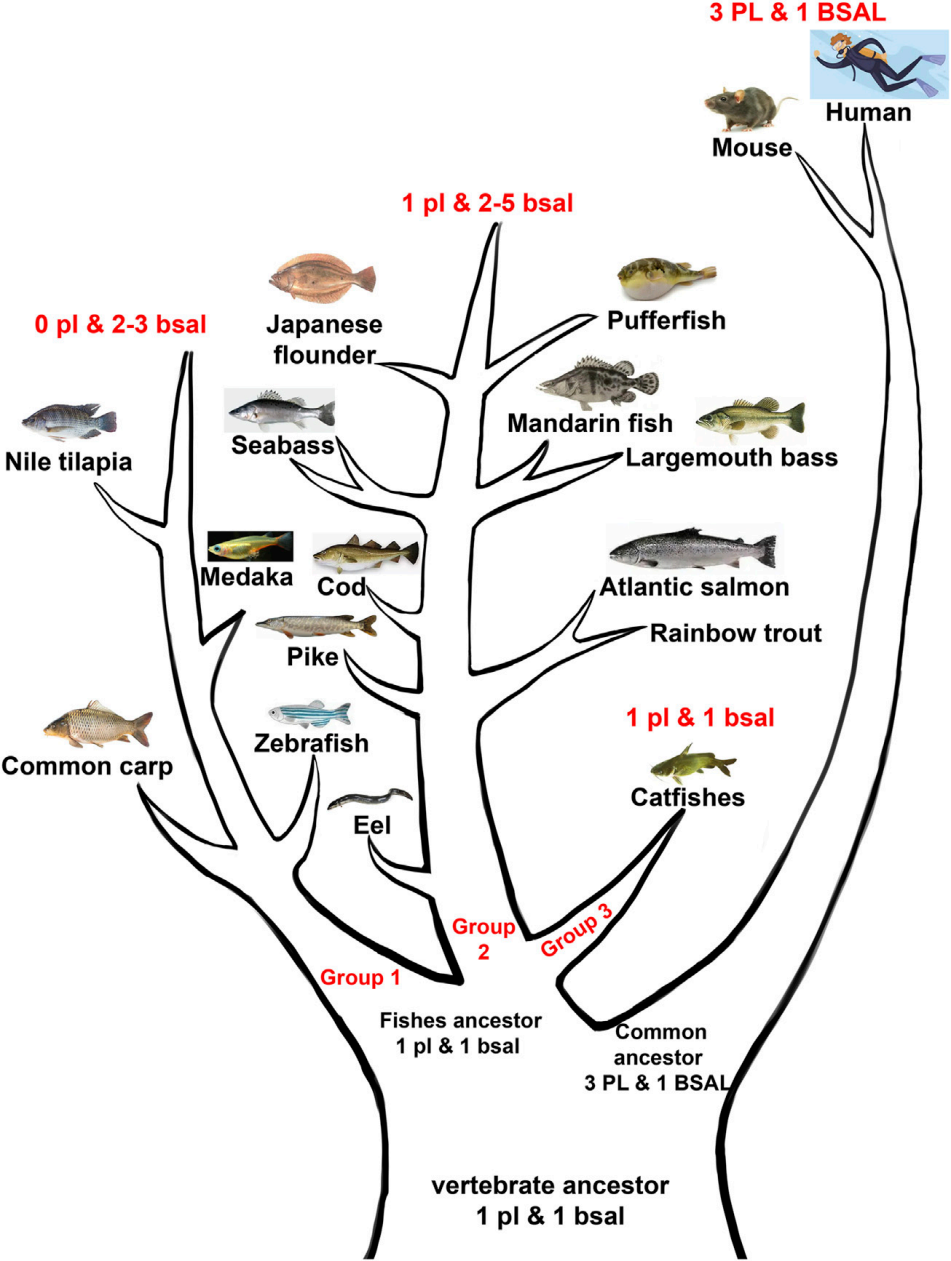
Abridged general view of digestive lipases during the evolution among mammals and fishes. The vertebrate ancestor might have one *pl* gene and 1 *bsal* gene, the fish ancestor retained the one *pl* gene and one *bsal* gene, then fish ancestor divided into three groups, the first group lost the *pl* gene but had two to three *bsal* gene copies (group 1), the second group had one *pl* gene and had two to five *bsal* gene copies (group 2), the third group retained the one *pl,* and 1 *bsal* (group 3). The common ancestor of mammals obtained other *PL* gene copies but retained one *BSAL* gene.

Moreover, the phylogenetic results demonstrated that the digestive lipase genes evolved independently between mammals and fishes (Figures 3, 4). The pancreatic lipase genes diverged into two groups between mammals and fishes, all *PL* genes of mammals were grouped together as the outgroup, and all *pl* genes of fish were grouped together (Figure 3). The same situation also could be seen in bile salt-activated lipase genes (Figure 4). The study suggested that the main digestive lipase gene might diverge into two types from the common ancestor of mammals and fishes. The spotted gar is a representative of holosteans, it diverged from the teleosts before the teleost genome duplication (TGD), and the genome of spotted gar has conserved from bony vertebrate ancestors (Amores et al., 2011; Pasquier et al., 2017; Martin & Holland, 2017). So the genome of the spotted gar was considered to be closest to vertebrate ancestors in this study. Therefore, we inferred that the *pl* gene and *bsal* gene both had the lipolytic ability in the vertebrate ancestors, and then the main digestive lipase gene evolved into different types between the mammals and fishes (Figure 9). The *PL* gene might play an important role in lipids digestion in mammals (Aloulou et al., 2014; Zhu et al., 2021), and the *PL* had occurred the gene tandem duplication in order to satisfy the lipids digestion ability (Figure 1). In contrast, the *bsal* might play a major function in lipids digestion in the ancestor of teleosts. Previous studies suggested that the massive gene deletions appeared after the third WGD (TGD) in teleost fishes and a large number of genes have been lost mostly through pseudogenization in rainbow trout after the fourth WGD (Berthelot et al., 2014; Inoue et al., 2015). So, we inferred that the *pl* gene might be absent (eg. zebrafish and medaka) or become a pseudogene (eg. rainbow trout and Atlantic salmon) with the WGD events that happened in the ancestor of some fish species. Although most of the fish species retained the *pl* gene, it might become an inactive *pl* gene, e.g., mandarin fish, seabass, and pufferfish. So, the *bsal* gene might be the main digestive lipase gene in fish and it is retained in all the teleost fishes. Moreover, the *bsal* gene might have extra two to five gene copies in order to satisfy the lipids digestion ability in all the teleost fishes. The *bsal* gene seemed to be more important in the teleost fishes, it might be related to the ability to hydrolyze polyunsaturated fatty acids (PUFAs) from triacylglycerol, but the pancreatic lipase can not hydrolyze those PUFAs (Bogevik et al., 2008; Anderson et al., 2018; Cai et al., 2017; Gilannejad et al., 2019, 2020). It also has been reported that *bsal* is far more efficient in hydrolyzing PUFAs from TAGs than *pl* (Sæle et al., 2010). As we all know, PUFAs are very important in fish larval development (Benedito-Palos et al., 2014; Fuentes-Quesada & Lazo, 2018; Arantzamendi et al., 2019). Thus, *bsal* might be a suitable digestive lipase for most fishes.

In order to get more information about the digestive lipases in fishes, 3D structures were used to analyze the digestive lipases. The results showed that the 3D structures of pancreatic lipases were conserved in mammals and fishes, and the lid domain, β-9 loop, and active site catalytic triad of Ser-His-Asp were present in all species (Figure 7). A previous study also indicated that the active sites and 3D structure of three *PL* genes were highly conserved in mammals (Aloulou et al., 2014). Although there had some controversies and doubts about pancreatic lipase in fish (Rønnestad et al., 2013; Yanes-Roca et al., 2018; Anderson et al., 2018), the 3D structure of pancreatic lipases in the present study demonstrated the pancreatic lipase really existed in fish, and the structures of pancreatic lipases were conserved from holosteans to teleosts. Therefore, we believed that the *pl* gene also really existed in fish, and the “*plrp*” gene in some fish species was a copy of *pl* gene, it might have a similar function to the *pl* gene. As we can see, the loop structure of bile salt-activated lipase appeared in the bile salt-binding site in most of the fish species (Figure 8). The function of this loop is related to the activation of Bsal (Holmes & Cox, 2011)*.* Specifically, the active site catalytic triad of Ser-His-Asp is centrally located within the Bsal structure and is partially covered by this loop (Hui & Howles, 2002). The bile salt activates Bsal by binding to the loop domain (a relatively short amino acid residue: GANFLXNYLY) (Wang et al., 1997; Murray et al., 2006; Holmes & Cox, 2011; Cai et al., 2017), bile salt trigger the lipase conformational changes and frees the active site allowing the access of water-insoluble substrates (lipids) (Karimi et al., 2010; Kurtovic et al., 2011). Although the amino acid residues of the bile salt-binding site (GANFLDNYLY) were correct in Bsal.1 of mandarin fish, the loop structure does not appear in the 3D structure. Lipases favor their interactions with TAGs at the interface through various interfacial phenomena and processes (Rueda-López et al., 2017), so the absence of loop structure might affect the lipolytic activity of Bsal.1 in mandarin fish. Meanwhile, the loop structure appeared in the Bsal.2 of the mandarin fish (see Supplementary Figure S4), but the amino acid residues of the bile salt-binding site had changed (DVAVLGNSLY) (Figure 6), which might also cause the bile salts could not bind to the loop domain. The special Bsal structure of the mandarin fish might be related to its special feeding habits, which only prey on other fish larvae but do not eat any zooplankton since the first feeding stage (Doi & Aoyama, 2004; Liang et al., 2008). It has been reported that the marine carnivorous fishes possess higher lipase activities than the herbivorous and omnivorous fish species (Zambonino-Infante et al., 2008; Wu et al., 2010; Heras et al., 2020), because they have adapted to ingest zooplankton rich in PUFAs from the larval stage (Rueda-López et al., 2017). The ancestor of the mandarin fish also is marine fish, which evolved in freshwater in East Asia (Li, 1991). So, we inferred that the structure and function of bile salt-activated lipase were changed in the ancestor of the mandarin fish in order to adapt to the freshwater environment.

In conclusion, we searched and identified the two types of important digestive lipase genes in mammals and fishes. The results showed that the *PL* gene occurred in the tandem duplication but the *BSAL* gene retained the one copy in mammals. In contrast, only one copy of *pl* gene was found in most of the fishes, the *pl* gene was absent or became a pseudogene in some fish species. However, the *bsal* gene existed in all fish species, even two to five copies of *bsal* gene were found in most fishes. Although the 3D structures of pancreatic lipase were conserved in all species, the amino acid sequences analysis showed that the Ile and Leu were absent in the lid domain and the β-9 loop might influence the lipolytic ability of pancreatic lipase in most of the fish species. But the key amino acid residues (bile salt-binding site and catalytic triad) and 3D structures of bile salt-activated lipase were conserved in mammals and fishes. By combining the phylogenetic results, we inferred that the main digestive lipase gene evolved into two types between mammals and fishes. The pancreatic lipase might play an important role in lipids digestion in mammals and the *PL* occurred the gene tandem duplication. In contrast, the bile salt-activated lipase might play a major function in the lipids digestion in the fishes and the *bsal* occurred gene duplication in most of the fishes.

## Data Availability

The datasets presented in this study can be found in online repositories. The names of the repository/repositories and accession number(s) can be found in the article/Supplementary Material.
