# Supplementary material for: Comparative Study of the Molecular Characterization, Evolution, and Structure Modeling of Digestive Lipase Genes Reveals the Different Evolutionary Selection Between Mammals and Fishes
Source: Front Genet. 2022 Aug 4;13:909091. doi: 10.3389/fgene.2022.909091 (PMC9386070; doi:10.3389/fgene.2022.909091)
Supplement: Supplementary file 1 [file Datasheet2.PDF]

```
                                               20                            40                            60                            80
PL - Human                 M--------- -----LPLWT LSL---LLGA VAGKEVCYER LGCFSDDSPW SGITERPLHI LPWSPKDVNT RFLLYTNENP 63
PLRP1 - Human              M--------- -----LIFWT ITLF--LLGA AKGKEVCYED LGCFSDTEPW GGTAIRPLKI LPWSPEKIGT RFLLYTNENP 64
PLRP3 - Human              M--------- -----LGIWI VAFL--FFGT SRGKEVCYER LGCFKDGLPW TRTFSTELVG LPWSPEKINT RFLLYTIHNP 64
Pl - Mouse                 M--------- -----LMLWT FAV---LLGA VAGREVCFDK LGCFSDDAPW SGTLDRPLKA LPWSPAQINT RFLLYTNENP 63
Plrp1 - Mouse              M--------- -----LILWT IPLF--LLGA AQGKEVCYDN LGCFSDAEPW AGTAIRPLKL LPWSPEKINT RFLLYTNENP 64
Plrp2 - Mouse              MPMDVRGCLF PSVQMLLCWL VSL---LLAT VGGKEVCYGH LGCFSNDKPW AGMIQRPSKI FPWSPEDIDT RFLLYTNENP 77
pl - spotted gar           M--------- -----LLSCV AVLF--FLGT VHTAEVCYGR LGCFTDDMPW AGTAERPIAK LPWSPEKISA RFLLYTRQNP 64
plrp - European eel        M--------- -----SVVWI LGLF--LLGT ASGGEVCYEN LGCFTDDVPW AGTTERPIAK LPWSPEKIGT RFMLFTRQNP 64
plrp - Northern pike       M--------- -----SQFWI LGLLGCLLGA AYAAEVCYSD LGCFKDDMPW AGTTERPITR LPWSPEKIGT RFLLYTRQNP 66
plrp - Atlantic cod        M--------- -----TQIWV LGAVLCLFGS AYADEVCYDG LGCFSDLPPW GGTKERPLAK LPWVPGMIET RFLLFTPKN- 65
pl - Yellow catfish        M--------- -----WLLWI SGLLHCLVGA VNGAEVCYDK LGCFSDEKPW SGTTERPIPC LPWSPEKINA RFILFTRENP 66
pl - Channel catfish       M--------- -----WILWI GGLLNCLFGA VYGAEVCFDK LGCFSDEFPW SGTTERPIAR LPWSPEKNNA HFLLFTRQNT 66
plrp - Largemouth bass     M--------- -----AQVWT LGLL-YLIGA SYTAEVCFNE LGCFDDLPPW GGTAQRPASV LPWHPEKIGT RFLLFTQKN- 64
plrp - Asian seabass       M--------- -----LQKWC LGLLCFLTGA SYAAEVCFDE LGCFNDLPPW GGTVQRPASV LPWNPEELGT RFLLFTQRN- 65
plrp - Mandarin fish       M--------- -----SQVWS LGLLCFLIGA SYAAEVCFND LGCFDDLPPW GGTAQRPASI LPWQPEKIGT RFLLFTQKN- 65
plrp - European seabass    M--------- -----SQ--S LGLLCFLIGA SYAAEVCFND LGCFSDLPPW GGTAQRPASV LPWPQEEIGT RFLLYTQKN- 63
plrp - Pufferfish          M--------- -----NTMWS LSVFCLLVSA TYATQVCFEG LGCFADGPPW GGTDQRPVSI LPSHPDEIGT RFLLFTQRN- 65
plrp - Japanese flounder   M--------- --------WC VGLFCFLIGA SYAAEVCFDE LGCFDDLAPW GGTPQRPTSI LPWNPDQIGT RFLLFTQKN- 62
Consensus                  M--------- -----LQLWX LGLL--LLGA XYGAEVCYDD LGCFSDXPPW GGTAXRPLSI LPWSPEKIGT RFLLXTQENP

                                              100                           120                           140                           160
PL - Human                 NNFQEVA-AD SSSISGSNFK TNRKTRFIIH GFIDKGEENW LANVCKNLFK VESVNCICVD WKGGSRTGYT QASQNIRIVG 142
PLRP1 - Human              NNFQILLLSD PSTIEASNFQ MDRKTRFIIH GFIDKGDESW VTDMCKKLFE VEEVNCICVD WKKGSQATYT QAANNVRVVG 144
PLRP3 - Human              NAYQEISAVN SSTIQASYFG TDKITRINIA GW--KTDGKW QRDMCNVLLQ LEDINCINLD WINGSR-EYI HAVNNLRVVG 141
Pl - Mouse                 DNYQLIT-SD ASNIRNSNFR TNRKTRIIIH GFIDKGEENW LSDMCKNMFR VESVNCICVD WKGGSRTTYT QATQNVRVVG 142
Plrp1 - Mouse              TAFQTLQLSD PSTIEASNFQ VARKTRFIIH GFIDKGEENW VVDMCKNMFQ VEEVNCICVD WKRGSQTTYT QAANNVRVVG 144
Plrp2 - Mouse              NNYQIISATD PATINASNFQ LDRKTRFIIH GFIDKGEEGW LLDMCKKMFQ VEKVNCICVD WKRGSRTEYT QASYNTRVVG 157
pl - spotted gar           NNHQEITADK VETILASNYR GNRTTRIITH GYIDKGDENW LIDMCKLMLQ IEDINCICVD WKKGGRTLYT QSAQNIRVIG 144
plrp - European eel        NNHQEITT-K EDILLASNYN GTRKTRFITH GYVDKGDENW LIDMCKLMLQ VEDINCICVD WKKGGRTLYT QSASNIRVIG 143
plrp - Northern pike       DAFQEIKADA A-ILKASNYN GDRMTRFIIH GFFDKGYENW LIDMCKTMLT VEDVNCICVD WRKGGLSLYT QSANNIRVVG 145
plrp - Atlantic cod        KYYHEIKA-D AKIMKVTNYN KLKATRFIIP GYLEAKDEDW AQHMCKDMLK WDSVNCIAME WKAGVRAPYA QAANNARVVA 144
pl - Yellow catfish        NSFQEIST-N ADVIAASKYQ ATRKTRFITH GFVDKGDENW LFDMCKLMLS IEDINCICVD WKSGSRSLYT QAANNIRVIG 145
pl - Channel catfish       DSYQEIST-N ANVIAGSTYR PSRKTRFITH GFIDKGDENW LLDMCKLMLS LEDINCICID WKSGSRTLYT QAANNIRVIG 145
plrp - Largemouth bass     RYYQEIKT-H QSI-QSSNYN GIRKTQFIIP GYLEKGDEDW PQEMCKVMLT KENVNCIAVE WKKGVRTQYA QAASNARVVS 142
plrp - Asian seabass       RYYQEIKT-D QTI-HASNYG GTRKTRFIIP GYLKKGDEDW PQEMCKVMLK WENVNCIAVE WKKGVKTQYA QAANNARVVA 143
plrp - Mandarin fish       RYYQEIKT-D QTI-QASNYS GMRKTRFIIP GYLKKRDEDW PQEMCKVMVK WENVNCIAVE WKKGVKTQYA QAANNARVVS 143
plrp - European seabass    TYYQEIKI-D QSI-LASNYN GKRKTRFIIP GYLEKGDEAW PQEMCKVMVK WDIVNCIAVE WKKGVKTQYA QAANNARVVA 141
plrp - Pufferfish          RYYQEIMA-D ETV-EASNYS GMRTSRFILP GYLLEGDEDW PQRMCKEMVK RENVNCIAVE WKKGVKTRYA QAANNIRVVA 143
plrp - Japanese flounder   RYYQEIWP-D KTI-HASNYN GLRKARFIIP GYLIEGEEDW PQEMCKDMVK WESVNCVAVE WTSGVKTKYA QAANNARVVA 140
Consensus                  NYYQEIKX-D ASIIXASNYN GDRKTRFIIH GYXDKGDENW LQDMCKVMLK VEDVNCICVD WKKGSRTLYT QAANNIRVVG

                                              180                           200                           220                           240
PL - Human                 AEVAYFVEFL QSAFGYSPSN VHVIGHSLGA HAAGEAGRRT NGTIGRITGL DPAEPCFQGT PELVRLDPSD AKFVDVIHTD 222
PLRP1 - Human              AQVAQMLDIL LTEYSYPPSK VHLIGHSLGA HVAGEAGSKT PG-LSRITGL DPVEASFEST PEEVRLDPSD ADFVDVIHTD 223
PLRP3 - Human              AEVAYFIDVL MKKFEYSPSK VHLIGHSLGA HLAGEAGSRI PG-LGRITGL DPAGPFFHNT PKEVRLDPSD ANFVDVIHTN 220
Pl - Mouse                 AEVALLVNVL QSDLGYSLNN VHLIGHSLGS HIAGEAGKRT FGAIGRITGL DPAEPYFQGT PEEVRLDPTD AQFVDAIHTD 222
Plrp1 - Mouse              AQVAQMIDIL VRNFNYSASK VHLIGHSLGA HVAGEAGSRT PG-LGRITGL DPVEANFEGT PEEVRLDPSD ADFVDVIHTD 223
Plrp2 - Mouse              AEIAFLVQVL STEMGYSPEN VHLIGHSLGS HVAGEAGRRL EGHVGRITGL DPAEPCFQGL PEEVRLDPSD AMFVDVIHTD 237
pl - spotted gar           AQMADMVTTF QTNFKQKPAD FHIIGHSLGA HAAGEAGRRL KG-LGRITGL DPAEPYFQGT PILVRLDPSD AVFVDVIHTD 223
plrp - European eel        AQMAYMIQLF QTVYQQRPES VHIIGHSLGA HCAGEAGRRT PN-LGRITGL DPAEPYFQGC PSLVRLDPSD AKFVDVIHTD 222
plrp - Northern pike       AQVAQMIEVF QDVYRQKPSM VHVIGHSLGA HAAGEVGRRI QK-LGRITGL DPAEPYFQGC SATVRLDPSD AMFVDVIHTD 224
plrp - Atlantic cod        AQVAHMINFL MGNYKQTADK FHIIGHSLGA HAAGEVGRLV PN-LARITGL DPVEPYFQGA SRDVRLDTSD AAFVDVIHTD 223
pl - Yellow catfish        AQIAYMISIF KENFQQDPES VHIIGHSLGA HLAAEAGRRT PR-LGRITGL DPAQPYFQGC PALVRLDPSD ALFVDVIHTD 224
pl - Channel catfish       AQIAYMISIF KESFQQNPEN VHIIGHSLGA HMAAEAGRRT PG-LGRITGL DPAEPYFQGC PPLVRLDPSD ALFVDVIHSD 224
plrp - Largemouth bass     AQVASMITFL MGNYKQTADK FHIIGHSIGA HAAGDVGSRI TG-ITRITGL DPAEPYFQDT DASVRLDTSD ATFVDVIHSD 221
plrp - Asian seabass       AQVASMISFL MGNYKQKADK FHIIGHSLGA HIAGDTGSRI TG-LARITGL DPSEPYFQDT NASVRLDTSD AAFVDVIHTD 222
plrp - Mandarin fish       AQVASMITFL MGNYKQNADK FHIIGHSLGA HAAGDAGSRI TG-LARITGL DPAEPYFQDT NASVSLDTSD ATFVDVIHSD 222
plrp - European seabass    AQVASMITFL MGNYRQKADV FHIIGHGIGA HAAGDAGSRI TG-LARITGL DPVEPYFQDT NAAVRLDTSD ATFVDVIHTD 220
plrp - Pufferfish          AQVAAMITFL MDNYKQTAGK FHMIGHSLGA HAAGDVGSRI PG-LARITGL DPSEPYFQGT SAAVSLDVTD ANFVDVIHTD 222
plrp - Japanese flounder   AQVASMIRFL MVNYKQKVDK FHIIGHSLGA HAAGDVGSRI PG-LPRITGL DPTEPYFQDT DASVRLDTSD AAFVDVIHTD 219
Consensus                  AQVAXMITFL MGNYKQXPDK VHIIGHSLGA HAAGEAGSRI PG-LGRITGL DPAEPYFQGT PAXVRLDPSD AXFVDVIHTD

                                              260                           280                           300                           320
PL - Human                 GAPIV-PNLG FGMSQVVGHL DFFPNGGVEM PGCKKNILSQ I-VDIDGIWE GTRDFAACNH LRSYKYYTDS IVNPDGFAGF 300
PLRP1 - Human              AAPLI-PFLG FGTNQQMGHL DFFPNGGESM PGCKKNALSQ I-VDLDGIWA GTRDFVACNH LRSYKYYLES ILNPDGFAAY 301
PLRP3 - Human              AARILF-ELG VGTIDACGHL DFYPNGGKHM PGCEDLITPL LKFNFNAYKK EMASFFDCNH ARSYQFYAES ILNPDAFIAY 299
Pl - Mouse                 AGPII-PNLG FGMSQTVGHL DFFPNGGIEM PGCQKNILSQ I-VDIDGIWE GTRNFAACNH LRSYKFYTDS IVNPTGFAGF 300
Plrp1 - Mouse              AAPLI-PFLG FGTNQMVGHF DFFPNGGQYM PGCKKNALSQ I-VDIDGIWS GTRDFVACNH LRSYKYYLES ILNPDGFAAY 301
Plrp2 - Mouse              SAPII-PYLG FGMSQKVGHL DFFPNGGKEM PGCQKNILST I-VDINGIWE GTRNFAACNH LRSYKYYASS ILNPDGFLGY 315
pl - spotted gar           SLPML-PYVG MGMSQAVGHL DFYPNGGEHM PGCDKNVISQ I-IDIDGIWE GTRDFVACNH LRSYKYYSDS ILNPEGFLGY 301
plrp - European eel        AKPMI-PYLG MGMAQAVGHL DFYPNGGEHM PGCDKNLISQ I-VDIDGIWE GTRDFVACNH LRSYKYYSDS ILNPEGFTGY 300
plrp - Northern pike       ILPFI-PYVG KGMSQAVGHI DFYPNGGEHM PGCDKNIAST I-VDIDGLWE GTRDFVACNH LRSYKYYNES IVSPKGFTGY 302
plrp - Atlantic cod        SLPFTKNKLG LGMSGNMGHI DFYPNGGELM PGCSKNS-GS P-SNLDGIWE GNVNFDGCNH ARAYQYYTES TVKAHGFVAY 301
pl - Yellow catfish        SLPVI-PYLG FGMSQAVGHL DFYPNGGESM PGCEKNAISQ I-VDIDGIWE GTRDFVACNH LRSYKYYTDS ILNPAGFLGY 302
pl - Channel catfish       ALPVI-PYLG FGMSEAVGHL DFYPNGGESM PGCEKNIISQ I-VDIDGIWE GTRDFVACNH LRAYKYYSDS ILNPKGFLGY 302
plrp - Largemouth bass     GLPF-KSKLG LGISQSVGHI DFYPNGGELM PGCSANK-GN P-TNLDAYWE GTKKFDACNH VRAYQYYSES VVKPQGFVAF 298
plrp - Asian seabass       GLPF-NSKLG LGMSQSVGHI DFYPNGGELM PGCSANK-GS P-TNLDAIWE GTMRFNDCNH VRAYQYYSES MVKPGGFVGF 299
plrp - Mandarin fish       GLPF-NSKLG LGMSQSVGHI DFYPNGGELM PGCSVNK-GN P-TDLDAIWE GTMKFDACNH VRAYQYYSES VVKPQGFVGF 299
plrp - European seabass    GLPF-NSKLG LGISQSVGHI DFYPNGGELM PGCTTNK-GK P-TDLDAIWE GTMKFDACNH VRAHQYYSES IVKSQGFVGF 297
plrp - Pufferfish          GLPF-DPKLG LGMSQSVGHI DFYPNGGQLM PGCSTNR-GD P-SDLDAIWL GDKKFDACNH VRAYEYYIES LEKPQGFMGY 299
plrp - Japanese flounder   GLPF-NSKLG LGMSQSVGHI DFYPNGGELM PGCSANK-GK P-SDLDAFWE GTKKFDGCNH VRAYQYYSES MVKPRGFVGY 296
Consensus                  GLPFI-PKLG XGMSQXVGHL DFYPNGGELM PGCSKNI-SQ I-VDIDGIWE GTRDFVACNH LRSYKYYSES IVNPDGFVGY
```

		340		360		380		400	
PL - Human	PCASYNVFTA	NKCFPCPSGG	-CPQMGHYAD	RYPGKT-NDV	GQKFYLDTGD	ASNFARWRYK	VSVTLSG-KK	V--TGHILVS	375
PLRP1 - Human	PCTSYKSFES	DKCFPCPDQG	-CPQMGHYAD	KFAGRT-SEE	QQKFFLNTGE	ASNFARWRYG	VSITLSG-RT	A--TGQIKVA	376
PLRP3 - Human	PCRSYTSFKA	GNCFFCSKEG	-CPTMGHFAD	RFHFKNMKTN	GSHYFLNTGS	LSPFARWRHK	LSVKLSGSEV	T--QGTVFLR	376
Pl - Mouse	SCSSYSLFTA	NKCFPCGSGG	-CPQMGHYAD	RYPGKT-SRL	YQTFYLNTGD	KSNFARWRYQ	VTVTLSG-QK	V--TGHILVS	375
Plrp1 - Mouse	PCASYRDFES	NKCFPCPDQG	-CPQMGHYAD	KFANNT-SVE	PQKFFLNTGE	AKNFARWRYR	VSLTFSG-RT	V--TGQVKVS	376
Plrp2 - Mouse	PCSSYEKFQH	NDCFPCPEQG	-CPKMGHYAD	QFEGKT-ATV	EQTFFLNTGD	SGNFTRWRYK	VSVTLSGAKK	L--SGYILVA	391
pl - spotted gar	PCNDDEAFKS	GSCFPCPQNG	-CPFMGHHAN	EFHVPK-GAE	KLKFYLNTGD	ARPFGRWQYK	VTVTVTGDRA	V--AGYIMVS	377
plrp - European eel	PCSDGGVFES	GRCFPCGDGA	-CPFMGHHAD	KFRRPN-GAE	KMKFYLNTAD	AKPFGRYRYK	VTVTIRGNRA	LLLTGTMSVA	378
plrp - Northern pike	PCSNQDVFDS	GKCFPCADQA	-CPIMGHYAD	TFHVPN-GQS	KLKFHLNTGE	AVPFSRYRYK	VSVNLDGSRS	V--SGIMKIA	378
plrp - Atlantic cod	PCPDKESFAA	GMCFPCTGA-	SCPLMGQSAI	KFNLTS-VPT	GTKFFLTTGK	KEPFGRYSYR	AKVLLDGSV-	WPNPGFMYIS	378
pl - Yellow catfish	PCSNQEMFES	GHCFPCDS-S	SCPFMGYHSD	KFNVTK-GVD	KMKFYLNTGD	ARPFSRYRYR	VTVTIDGSRT	SL--GYFKVA	378
pl - Channel catfish	PCSNQNMFES	GHCFPCASNS	SCPFMGHHAD	QFKVPN-GVD	KMKFQLNTGY	ARPFSRYRYK	VTVTIDGSQT	NK--GYFKVA	379
plrp - Largemouth bass	PCSDKDSFAA	GKCFPCADN-	KCPLMGHQAD	KFTVTD-GIL	MTKYFLNTGS	SKSFSRYSYK	VTVTLNGPS-	WPNPGFMYVA	375
plrp - Asian seabass	PCSDKDSFAA	GKCFPCANN-	KCPLMGHRAD	RFTVTD-GIS	KTKYFLNTGG	SEPFGRYSYK	MTVTLDGSK-	WPNPGFMFVA	376
plrp - Mandarin fish	PCSDKDSFAA	GKCFPCADN-	KCPLMGHHAD	KFTVTD-GIS	KTKFFLNTGS	SKPFGCYSYR	VMLTLDGPR-	WPNPGSMYVA	376
plrp - European seabass	PCSDKGSFDD	GKCFPCADD-	KCPLMGHYAE	RFTLTD-GIS	KMKYFLNTGS	LNPFGRYSYR	ATVTLDGPR-	WPNPGFMYVA	374
plrp - Pufferfish	PCPNKDSFAD	GKCFPCGHT-	ECPLMGHRAD	RFTGTE-DTS	ITKYFLTTGS	KAPFRRYSYR	VSVTLAGPI-	LPNVGFMFVA	376
plrp - Japanese flounder	PCSNKDSFAA	GKCFPCENG-	KCPLMGHDAD	RFTVTD-GVS	KTNYFLNTGA	SKPFGRYSYR	VTLTLDGPS-	WANLGLMFVA	373
Consensus	PCSDKDSFAA	GKCFPCADNG	-CPLMGHYAD	XFTVTX-GXS	KTKFFLNTGD	AKPFGRYRYK	VTVTLDGSR-	W--XGFMKVA	

		420		440		460		480	
PL - Human	LFGNKGNSKQ	YEIFKGTLKP	DSTHSNEFDS	DVDVGDLQMV	KFIWYNNVIN	PTLPRVGASK	IIVETNVGK-	QFNF-----C	449
PLRP1 - Human	LFGNKGNTHQ	YSIFRGILKP	GSTHSYEFDA	KLDVGTIEKV	KFLWNNNVIN	PTLPKVGATK	ITVQKGEEKT	VYNF-----C	451
PLRP3 - Human	VGGAVRKTGE	FAIVSGKLEP	GMTYTKLIDA	DVNVGNITSV	QFIWKKHLFE	DSQNKLGAEM	VINTSGKYGY	KSTF-----C	451
Pl - Mouse	LFGNGGNSKQ	YEVFKGSLQP	GTSHVNEFDS	DVDVGDLQKV	KFIWYNNVIN	PTLPKVGASR	ITVERNDGR-	VFNF-----C	449
Plrp1 - Mouse	LFGSNGNTRQ	CDIFRGIIKP	GATHSNEFDA	KLDVGTIEKV	KFLWNNHVVN	PSFPKVGAAK	ITVQKGEERT	EHNF-----C	451
Plrp2 - Mouse	LYGCNGNSKQ	YEVFKGSLQP	EARYIRDIDV	DVNVGEIQKV	KFLWNNKVIN	LFRPTMGASQ	ITVQRGKDGK	EFNF-----C	466
pl - spotted gar	VFGTNGNTKQ	YQIMKGTLRP	GRTYIGFIDT	ELDVGDVTKV	KFLWSNSQIN	PLLPTFGGET	VVVQRGEDGK	MFQF-----C	452
plrp - European eel	IYGTQGNTRQ	YQIRKGLLKP	GNTYEAYIDT	ETDAGEVTKM	KFIWDNSVIN	PLFPKLGAEK	IVLQRGKDRR	VFSF-----C	453
plrp - Northern pike	LYGEQGNTRQ	YTVHSGRLIP	GKTYEVFVDA	ETDIGEVTRM	KFLWNNNIIN	PLHPKLGAAR	IELQRGVDRK	VYQF-----C	453
plrp - Atlantic cod	LKGDREETES	IQLHVGMLSP	GF-YELLINT	DKDVGEIKEM	TFQWNNHIFN	PIKPTYSASR	IELVRGKDNK	TYNF-----C	452
pl - Yellow catfish	LYGVNGNTRQ	YEIHKGTLSP	GRTYELLIDV	EKEIDELTFV	KFIWNNKVLN	PLLPKFGATN	IVVQRGRDRK	MFKF-----C	453
pl - Channel catfish	LYGVDGNTRQ	YQNYNGTLSP	GSTYELLIDV	EKDVDELTHV	KFIWNNDALI	PLRPKFGATQ	IIVQRGRDRK	TFKF-----C	454
plrp - Largemouth bass	FVGDKDSTKE	YKIYEGILRP	GRNYERLIDA	EVDMGDVTEV	KFRWNNHILN	PLRPKYGASK	VKLQRGKDKK	IVFF-----C	450
plrp - Asian seabass	LTGNSDSTKE	YQLHVGTLMP	GSTYEMLFNA	EVDVGDVTEV	KFRWNNHIFN	PMKPKYGASK	VELQRGKDNK	TVLF-----C	451
plrp - Mandarin fish	FVGDNDSTKE	YQLYVGKLVA	GWTYEVLINT	EVDLVDVTEV	KFRWNNYIFN	PMKPKYGASK	VELQRGKDQK	IVLF-----C	451
plrp - European seabass	LTGDNDSTKE	HQLYVGTMVP	GRTYEVLIDA	EVDVGDVTEV	KFRWNNHIFN	PMNPKYGASK	VELQRGKDKK	IFSF-----C	449
plrp - Pufferfish	LVGKYGSTKE	HQLHVGTLIS	GRTYELLLDA	QLDVGDVTEV	QFRWNNHIID	PLRPKFGAEK	VVLQRGKDKE	IRSF-----C	451
plrp - Japanese flounder	FTGDGDSTEE	YQLHVGTMVP	GRTYELLLNA	EVDMGDVTEV	KFRWNNHIFN	PLKPKYGASK	VELLRGKDNK	TVVF-----C	448
Consensus	LXGDNGNTKQ	YQIHXGTLKP	GRTYELLIDA	EVDVGDVTEV	KFXWNNHIIN	PLLPKYGASK	IELQRGKDRK	XFNF-----C	

		500		520		540		560	
PL - Human	SPETVREEVL	LTL-------	---TPC----	----------	----------	----------	----------	----------	465
PLRP1 - Human	SEDTVREDTL	LTL-------	---TPC----	----------	----------	----------	----------	----------	467
PLRP3 - Human	SQDIMGPNIL	QNL-------	---KPC----	----------	----------	----------	----------	----------	467
Pl - Mouse	SQETVREDVL	LTL-------	---SPC----	----------	----------	----------	----------	----------	465
Plrp1 - Mouse	SEETVREDIL	LTL-------	---LPC----	----------	----------	----------	----------	----------	467
Plrp2 - Mouse	SSNTVHEDVL	QSL-------	---YPC----	----------	----------	----------	----------	----------	482
pl - spotted gar	GRGQVRKDIL	QTL-------	---DAC----	----------	----------	----------	----------	----------	468
plrp - European eel	SSETVREEVL	QTV-------	---PLC----	----------	----------	----------	----------	----------	469
plrp - Northern pike	GKKMVGEDVL	QTL-------	---DPC----	----------	----------	----------	----------	----------	469
plrp - Atlantic cod	GGDRVGEKVI	QSV-------	---PPC----	----------	----------	----------	----------	----------	468
pl - Yellow catfish	GKDWVRENVL	QTL-------	---STC----	----------	----------	----------	----------	----------	469
pl - Channel catfish	GSEQVQENVL	QTL-------	---CPC----	----------	----------	----------	----------	----------	470
plrp - Largemouth bass	GTEKVAENKI	QSV-------	---FPC----	----------	----------	----------	----------	----------	466
plrp - Asian seabass	GTENVVENAV	QSV-------	---LPC----	----------	----------	----------	----------	----------	467
plrp - Mandarin fish	GTENVVENAI	QSV-------	---LPC----	----------	----------	----------	----------	----------	467
plrp - European seabass	GTGNVVENAI	QSV-------	---LLC----	----------	----------	----------	----------	----------	465
plrp - Pufferfish	GRANVAENEV	QSV-------	---LLC----	----------	----------	----------	----------	----------	467
plrp - Japanese flounder	GTYNVEENAI	QSV-------	---FPC----	----------	----------	----------	----------	----------	464
Consensus	GTETVRENVL	QTL-------	---LPC----	----------	----------	----------	----------	----------	

		580		
PL - Human	----------	----------	--------	465
PLRP1 - Human	----------	----------	--------	467
PLRP3 - Human	----------	----------	--------	467
Pl - Mouse	----------	----------	--------	465
Plrp1 - Mouse	----------	----------	--KTSDTM	473
Plrp2 - Mouse	----------	----------	--------	482
pl - spotted gar	----------	----------	--TF---L	471
plrp - European eel	----------	----------	--------	469
plrp - Northern pike	----------	----------	--GL---Q	472
plrp - Atlantic cod	----------	----------	--KT----	470
pl - Yellow catfish	----------	----------	--Q-----	470
pl - Channel catfish	----------	----------	--Q-----	471
plrp - Largemouth bass	----------	----------	--QA----	468
plrp - Asian seabass	----------	----------	--QV----	469
plrp - Mandarin fish	----------	----------	--QA----	469
plrp - European seabass	----------	----------	--QA----	467
plrp - Pufferfish	----------	----------	--EV----	469
plrp - Japanese flounder	----------	----------	--------	464
Consensus	----------	----------	--------	
