# Supplementary material for: Comparative Study of the Molecular Characterization, Evolution, and Structure Modeling of Digestive Lipase Genes Reveals the Different Evolutionary Selection Between Mammals and Fishes
Source: Front Genet. 2022 Aug 4;13:909091. doi: 10.3389/fgene.2022.909091 (PMC9386070; doi:10.3389/fgene.2022.909091)
Supplement: Supplementary file 2 [file Datasheet4.PDF]

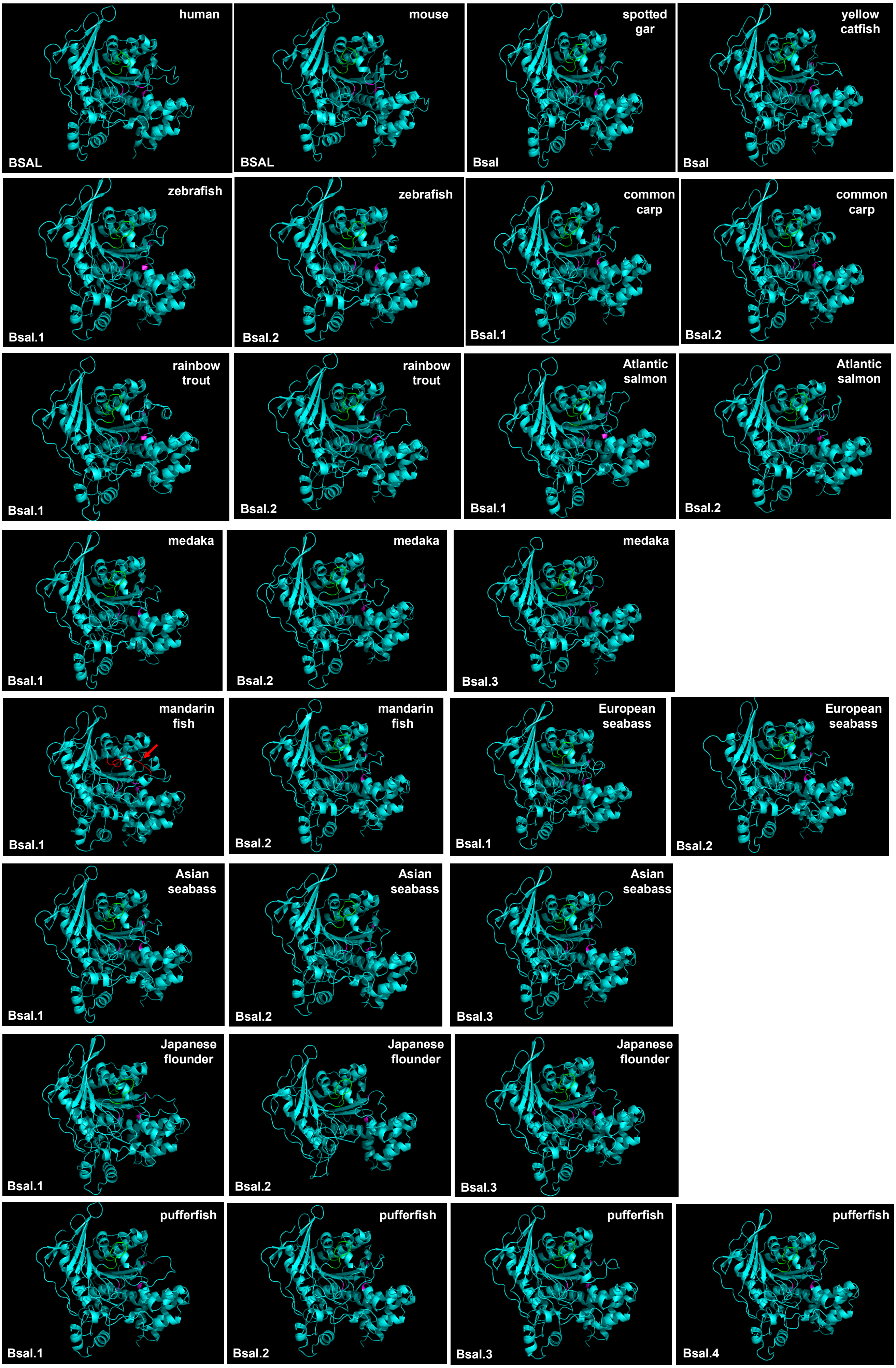

Supplementary Fig. 1 3-D structures of bile salt-activated lipases in human, mouse and fishes. The region of amino acid residues in bile salt-binding site (GANFLXNYLY) were marked with green. Active site triad residues Ser, Asp, and His were marked with pink. The red arrow represent the no loop structure existed in mandarin fish.
