# Supplementary material for: Comparative Study of the Molecular Characterization, Evolution, and Structure Modeling of Digestive Lipase Genes Reveals the Different Evolutionary Selection Between Mammals and Fishes
Source: Front Genet. 2022 Aug 4;13:909091. doi: 10.3389/fgene.2022.909091 (PMC9386070; doi:10.3389/fgene.2022.909091)
Supplement: Supplementary file 3 [file Datasheet3.PDF]

		20		40		60		80	
BSAL - Human	MGRLQL----	----------	----------	----------	------VVLG	LTCCWAVAS-	----AAKLGA	VYT-------	28
Bsal - Mouse	MGRLEV----	----------	----------	----------	------LFLG	LTCCLAAAC-	----AAKLGA	VYT-------	28
bsal - Spotted gar	MG--------	----------	----------	----------	---SWGTLLA	FALCVGCAS-	----AATLGV	VYT-------	27
bsal.1 - European eel	MT--------	----------	----------	----------	---VLEIAVV	VALCLGSAS-	----AASLGP	VLT-------	27
bsal.2 - European eel	MT--------	----------	----------	----------	---VLEIAVV	VALCLGSAS-	----AASLGP	VLT-------	27
bsal.1 - Northern pike	MEEHHIDAVR	NWPTLTTVKE	VQQFLGFSNY	YRRFIPGFSQ	VAALITYLLK	GGGDPVAMDR	RDRGARTLGP	TRCPTSMTRR	80
bsal.2 - Northern pike	MNYIELQKEL	NAIGRVIAYC	GNGRSLLWYK	RPASRSLWAS	IMKMLGILVA	VALFMGPAS-	----AATLGV	VYT-------	68
bsal.1 - Atlantic cod	MK--------	----------	----------	---FLLVTG-	---VLCLWLA	PAW-------	----CASLGV	VQT-------	27
bsal.2 - Atlantic cod	M---------	----------	----------	----------	MMKTLGVLVV	VALSWGAAS-	----AESLGT	VFT-------	29
bsal.3 - Atlantic cod	M---------	----------	----------	----------	-MRALGLSVA	IGLFLEMAS-	----GASLGT	VYT-------	28
bsal.4 - Atlantic cod	M---------	----------	----------	----------	-MRALWLLVA	IGLLLEMAS-	----GASLGT	VYT-------	28
bsal.5 - Atlantic cod	ME--------	----------	----------	----------	---PLAILLA	VGLFLGATS-	----AASLGV	VET-------	27
bsal - Yellow catfish	M---------	----------	----------	----------	AV--LGILAV	LGFFLSAVN-	----ASSLGA	VYT-------	27
bsal - Channel catfish	M---------	----------	----------	----------	VG--LGILAV	LGLFMSAAN-	----ASSLGV	VYT-------	27
bsal.1 - Atlantic salmon	M---------	----------	----------	----------	-MKTLGILVA	SALFLGSAS-	----AATLGV	VYT-------	28
bsal.2 - Atlantic salmon	M---------	----------	----------	---------T	VMKTLGILVS	AALFLGSAS-	----AATLGV	VYT-------	30
bsal.1 - Rainbow trout	M---------	----------	----------	----------	-MKTLGILVA	SALFLGSAS-	----AATLGV	VYT-------	28
bsal.2 - Rainbow trout	M---------	----------	----------	---------T	VMKTLGILVS	AALFLGSAS-	----AATLGV	VYT-------	30
bsal.1 - Largemouth bass	MLF-------	----------	GGGVSFISLT	DNEPGLHSAV	MMAKLGILVA	VAVFLETVS-	----AASLGV	VYT-------	51
bsal.2 - Largemouth bass	MLF-------	----------	GGGVSFISLT	DNEPGLHSAV	MMAKLGILVA	VAVFLETVS-	----AASLGV	VYT-------	51
bsal.3 - Largemouth bass	M---------	----------	----------	---MLLV---	--FMFGFLLN	SAS-------	----AAKLGV	VKT-------	25
bsal.1 - Asian seabass	M---------	----------	----------	---LLVV---	--VLFGIWLN	SAS-------	----AAKLGV	VQT-------	25
bsal.2 - Asian seabass	M---------	----------	----------	----------	-MLKVGVLVA	VAVFLETVS-	----GTSLGV	VYT-------	28
bsal.3 - Asian seabass	ME--------	----------	----------	----------	---KLKILLA	VVLSLGTAS-	----AASLGV	VQT-------	27
bsal.1 - Mandarin fish	M---------	----------	----------	----------	-MAKLGILVA	VAVFLETVS-	----AATLGV	VYT-------	28
bsal.2 - Mandarin fish	M---------	----------	----------	---MLLL---	---LFGVWLN	SAS-------	----AANLGV	VQT-------	24
bsal.1 - European seabass	MG--------	----------	----------	----------	---KLGILVA	VAVFLETVS-	----ATSLGT	VYT-------	27
bsal.2 - European seabass	MD--------	----------	----------	---CVCVCVC	VCVCVCVCVC	VCV-------	----CELYSN	VRI-------	31
bsal.1 - Pufferfish	MK--------	----------	----------	---MLLV---	--VLVTLWLD	AAT-------	----SAKLGV	VQT-------	26
bsal.2 - Pufferfish	M---------	----------	----------	----------	--AMFGIVVA	VAVFLETVS-	----ATSLGV	VYT-------	27
bsal.3 - Pufferfish	M---------	----------	----------	----------	--AMFGIVVA	VAVFLETVS-	----ATSLGV	VYT-------	27
bsal.4 - Pufferfish	ME--------	----------	----------	----------	---KQKSLLV	LALFVTTAS-	----AASLGL	VQT-------	27
bsal.1 - Japanese flounder	M---------	----------	----------	----------	-MARLGLLVA	FAVFLEVVS-	----GASLGV	VYT-------	28
bsal.2 - Japanese flounder	M---------	----------	----------	----------	--ARLGLLVA	FAVFLEVVS-	----GASLGV	VNT-------	27
bsal.3 - Japanese flounder	ME--------	----------	----------	----------	---KVKILLA	VVLSLGTAS-	----AASLGV	VQT-------	27
bsal.1 - Zebrafish	M---------	----------	----------	----------	AM--LGVFIS	AVLFLGTAQ-	----GASLGA	VLT-------	27
bsal.2 - Zebrafish	M---------	----------	----------	----------	MM-MLGVFIS	AALFLGTAQ-	----GASLGA	VLT-------	28
bsal.1 - Medaka	MK--------	----------	----------	---MMLV---	--VLLAVCLN	SAL-------	----AAKLGV	VQT-------	26
bsal.2 - Medaka	M---------	----------	----------	----------	--AMLGILAA	VAVFLQTVS-	----AASLGV	VNT-------	27
bsal.3 - Medaka	M---------	----------	----------	-------PPS	LTMMFRILAA	FAVFLQAVS-	----ATSLGI	VNT-------	32
bsal.1 - Common carp	M---------	----------	----------	----------	MMPMLGVFIS	VALILGTAN-	----AASLGS	VYT-------	29
bsal.2 - Common carp	M---------	----------	----------	----------	TMAMLGVFIS	VALILGTAN-	----AASLGA	VYT-------	29
bsal.1 - Tilapia nilotica	M---------	----------	----------	----------	-MLKLGILFA	VAVFLETAS-	----AASLGV	VQT-------	28
bsal.2 - Tilapia nilotica	M---------	----------	----------	----------	-MLKLGILFA	VALFLETAS-	----AASLGV	VQT-------	28
bsal.3 - Tilapia nilotica	M---------	----------	----------	----------	-MLKLGILFA	VALFLETAS-	----AASLGV	VQT-------	28
Consensus	M---------	----------	----------	----------	-M-KLGILVA	VALFLGTAS-	----AASLGV	VYT-------	

```
                                                   100        120        140        160
                                                     |          |          |          |
BSAL - Human                ---------- --------EG GFVEGVNKKL GLLG-DSVDI FKGIPFAAPT KALENPQPHP GWQGTLKAKN FKKRCLQATI 89
Bsal - Mouse                ---------- --------EG GFVEGVNKKL SLLGGDSVDI FKGIPFAT-A KTLENPQRHP GWQGTLKATN FKKRCLQATI 89
bsal - Spotted gar          ---------- --------EG GMVEGTHKR- KGL-FRSMDI FKGIPFADRP GRFENPKPHP GWSGVLKATE FRKRCLQITF 87
bsal.1 - European eel       ---------- --------EG GMVDGENKHV -GL-FRYMDV FKGIPFAAQP QRFEKPQPHP GWEGVLKAKK FARRCMQVNL 87
bsal.2 - European eel       ---------- --------EG GMVEGKNIHV -GL-FRYMDV FKGIPFAAQP QRFEKPQPHP GWEGVLKAKK FARRCMQVNL 87
bsal.1 - Northern pike      RVDDAPVLPA SCIMGAGGEG GMVKGKNHGM GFLSGRSVDV FKGIPFADPP ERFEKPKPHR GWDGVLNAYE LKERCLQVTL 160
bsal.2 - Northern pike      ---------- --------EG GMVEGNNINT DGL-FRTMDV FRGIPFADKP GKFEKPKRHP GWDGVLKATE FKPRCMQVTL 129
bsal.1 - Atlantic cod       ---------- --------EG GKVKGKNQNM GLL--HSVDV FKGIPFADVP GRWELPKRHP GWDGVLQAIK YQKRCLQVTL 87
bsal.2 - Atlantic cod       ---------- --------EG GLVEGTNIRL -GY-RRHMDV FKGVPFAAIP GRFEKPRRHP GWEGVLKATE FMKRCLQLNL 89
bsal.3 - Atlantic cod       ---------- --------EG GLVEGKNIRL -GY-KHHMDV FKGIPFAGMP GRFEKPERHP GWDGILKAFE YRPRCLQLNL 88
bsal.4 - Atlantic cod       ---------- --------EG GLVKGKNIRL -GY-KHHMDV FKGIPFAGMP GRFEKPERHP GWDGILKAFE YRPRCLQLNL 88
bsal.5 - Atlantic cod       ---------- --------EG GSVQGTNVRL -GL-RRSMDV FKGIPFAAKP GTFEKPKPHP GWTNTLKATK FAKRCLQKSM 87
bsal - Yellow catfish       ---------- --------EG GLVEGKNKNL -GL-FRSMDV FKGIPFAAQP VRFQKPTPHP GWSGVLKAND FPKRCLQLNL 87
bsal - Channel catfish      ---------- --------EG GLVEGKNHFV -GL-FRYMDV FKGVPFAAQP GRFQKPIPHR GWNGVLKAND FPKRCLQLDL 87
bsal.1 - Atlantic salmon    ---------- --------EG GMVQGKKVNS DGL-LRTMDV FKGIPYADKP GVFEKPKRHP GWDGVLKATE FKPRCMQLNL 89
bsal.2 - Atlantic salmon    ---------- --------EG GMVQGKNINS DGL-FRTMDV FKGIPFADKP GKFEKPKRHP GWDGVLKATK FKPRCMQLNL 91
bsal.1 - Rainbow trout      ---------- --------EG GMVQGKKVNS DGL-LRTMDV FKGIPYADKP GVFEKPKRHP GWDGVLKATE FKPRCMQLNL 89
bsal.2 - Rainbow trout      ---------- --------EG GMVQGKNINS DGL-FRTMDV FKGIPFADKP GKFEKPKRHP GWDGVLKATK FKPRCMQLNL 91
bsal.1 - Largemouth bass    ---------- --------EG GMVQGENIRL -GF-RRHMDV FRGIPFADIP GRFEKPKRHP GWDGVLKATE YRKRCLQLNL 111
bsal.2 - Largemouth bass    ---------- --------EG GMVQGENIRL -GF-RRHMDV FRGIPFADIP GRFEKPKRHP GWDGVLKATE YRKRCLQLNL 111
bsal.3 - Largemouth bass    ---------- --------EG GMVKGKSHGM GLF--RSVDV FKGIPFADVP GKWEKPKPHP GWSGTLKATK YRDRCLQVTL 85
bsal.1 - Asian seabass      ---------- --------EG GQVEGKSHRM GLF--RSVDV FKGIPFADVP GKWEKPKPHP GWSGILKATK YRDRCLQVTL 85
bsal.2 - Asian seabass      ---------- --------EG GMVQGENIRL -GF-RRHMDI FKGIPFADIP GRFEKPKRHP GWDGVLKATE YRKRCLQVNL 88
bsal.3 - Asian seabass      ---------- --------EG GSVRGQNIPL -GL-FRSVDV FKGIPFAAKP GTFEKPKPHP GWDGVLKATK FANRCLQISM 87
bsal.1 - Mandarin fish      ---------- --------EG GMVQGQNIRL -GF-RRHMDV FKGIPFAAIP GRFEKPKSHP GWDGILQATE FRKRCLQVNL 88
bsal.2 - Mandarin fish      ---------- --------EG GQVEGQSHRM GLF--STVDV FKGIPFADVP GKWEKPKPHP GWSGILKATK YRDRCLQVTL 84
bsal.1 - European seabass   ---------- --------EG GMVQGENIRL -GF-RRHMDI FKGIPFADIP GRFEKPKRHP GWDGVLKTTE YRPRCMQVNL 87
bsal.2 - European seabass   ---------- --------KV QFITVMITQT GLF--TVAEV PNPIPALATR RGWSALRTCL LIPGILKAIK YRDRCLQVTL 91
bsal.1 - Pufferfish        ---------- --------EA GLVEGRTHRM GLF--RTVEV FKGVPFADVP RRWEKPKAHP GWSGILKATK AQDRCLQVNL 86
bsal.2 - Pufferfish        ---------- --------EG GMVEGDNIRL -GF-RRHMDI FKGIPFADMP GRFEKPKRHP GWDGVLKAKE YRNRCLQVNL 87
bsal.3 - Pufferfish        ---------- --------EG GMVEGDNIRL -GF-RRHMDI FKGIPFADMP GRFEKPKRHP GWDGVLKAKE YRNRCLQVNF 87
bsal.4 - Pufferfish        ---------- --------EG GRVQGQNIPL -DV-VRSVDV FKGIPFAAKP GLFEKPKPHP GWDGILQATR FAEQCLQIND 87
bsal.1 - Japanese flounder ---------- --------EG GMVQGENIRL -GF-RRHMDV FRGVPFADIP GRFEKPKRHP GWDGVLKATE FRKRCLQVNL 88
bsal.2 - Japanese flounder ---------- --------EG GMVQGENIRL -GL-RRHMDV FRGVPFADIP GRFEKPKRHP GWDGILKATE FRKKCLQLKF 87
bsal.3 - Japanese flounder ---------- --------EG GSVQGKNIPL -RL-FRSVDV FKGIPFAAKP GTFEKPNPHP GWDGVLKATK FADRCLQISM 87
bsal.1 - Zebrafish         ---------- --------EG GMVQGKSRSV -GL-FRYMDT FKGIPFAAPP KRFEKPVAHP GWEGVLKTTD YRKRCLQLNL 87
bsal.2 - Zebrafish         ---------- --------EG GMVQGKSHSV -GL-LRYMDV FKGIPFAAPP GRLQKPVPHP GWEGVLKATE YSKRCIQLNL 88
bsal.1 - Medaka            ---------- --------EG GLVEGESIRM SLF--RTVDV FKGVPFADVP GKWEKPKPHP GWDGVLRATK YRDRCLQVTL 86
bsal.2 - Medaka            ---------- --------EG GMVEGKTIRL -GL-LRTMDV FRGVPFAAVP GRFEKPKPHP GWDGVMKATA FKSRCLQLDI 87
bsal.3 - Medaka            ---------- --------EG GLVEGENILL -EN-SRYVDV FKGIPFADFP GRFEKPKRHP GWDGVLKATK FQRRCLQLDL 92
bsal.1 - Common carp       ---------- --------EG GMVQGKSHSV -GL-FRYMDV FKGIPFAAPP GRLEKPVPHP GWDGVLKATD YRMRCMQLNL 89
bsal.2 - Common carp       ---------- --------EG GMVQGENHNV -GL-FRYMDV FKGIPFAAPP GRLEKPVPHP GWSGVLKATD YRKRCMQLNL 89
bsal.1 - Tilapia nilotica  ---------- --------EG GMVEGETISL -GL-SRSMDI FKGIPFADIP GRFEKPKRHP GWGGVLKATQ YSDECLQLNS 88
bsal.2 - Tilapia nilotica  ---------- --------EG GKVEGKSISL -GS-GRSMDI FLGVPFADAP GTFEKPRPHR GWDGILQAKD YKPRCLQLTV 88
bsal.3 - Tilapia nilotica  ---------- --------EG GKVEGKSISL -GS-GRSMDI FLGVPFADAP GTFEKPRPHR GWDGILQAKD YKPRCLQVNL 88
Consensus                   ---------- --------EG GMVEGKNIRL -GL-FRSMDV FKGIPFADXP GRFEKPKPHP GWDGVLKATE FRKRCLQLNL
```

		180		200		220		240	
BSAL - Human	TQDSTYGDED	CLYLNIWVPQ	GRKQVSRDLP	VMIWIYGGAF	LMGSGHGANF	LNNYLYDGEE	IATRGNVIVV	TFNYRVGPLG	169
Bsal - Mouse	TQDNTYGQED	CLYLNIWVPQ	GRKQVSHNLP	VMVWIYGGAF	LMGSGQGANF	LKNYLYDGEE	IATRGNVIVV	TFNYRVGPLG	169
bsal - Spotted gar	TQTSTRGSED	CLYLNIWVPQ	GS-KVSTGLP	VMVYIYGGAF	LVGGSQGANF	LENYLYSGEE	IADRGKVIVV	TANYRVGTLG	166
bsal.1 - European eel	IQTETRGSED	CLYLNIWVPQ	GR-SVSTGLP	VMVYLFGGGF	LVGGSMGANF	LDNYLYSGEE	IADRGKVIVV	TVGYRVGTLG	166
bsal.2 - European eel	IQTETRGSED	CLYLNIWVPQ	GR-SVSTGLP	VMVYLYGGAF	LVGGSMGANF	LDNYLYSGEE	IADRGNVIVV	TLGYRVGTLG	166
bsal.1 - Northern pike	MQSSTHGSKD	FLYLNIFVPQ	NKGQVSMGLP	VMVYLFGGAF	LLRASNDVSF	LGNSLYNGKE	IADRGKVIIV	TVNYRVGVLG	240
bsal.2 - Northern pike	LQNDVRGQED	CLYLNIWVPQ	GR-SVSTGLP	VMVWIYGGGF	LVGGSSGANF	LNNYLYDGEE	IANRGKVIVV	TLNYRVGTLG	208
bsal.1 - Atlantic cod	LQLSSQGSED	CLYLNIHVPQ	GGLSLSRGLP	VMVYIFGGAF	LLGASNDVPF	LGNSLYDGNE	MADRGGVIVV	SVNYRVGSLG	167
bsal.2 - Atlantic cod	LQTGTRGSED	CLYLNIWVPH	GR-EVSTGLP	VMVWIYGGGF	LVGGSMGANF	LDNYLYSGQE	IADRGDVIVV	TIGYRVGALG	168
bsal.3 - Atlantic cod	IMTSTRGSED	CLYLNIWVPH	HR-SVSTKLP	VMVWIYGGAF	LWGGSMGANF	LNNYLYSGQE	IADRGDVIVV	TVAYRLGPLG	167
bsal.4 - Atlantic cod	IMTSTRGSED	CLYLNIWVPH	HR-SVSTNLP	VMVWIYGGAF	LVGGSMGANF	LNNYLYSGQE	IADRGDVIVV	TVAYRLGPLG	167
bsal.5 - Atlantic cod	LQTSSFGVED	CLHLNIWVPH	GL-YVSSNLP	VMVWFYGGGF	LAGGSMGPNF	FDNYLYGGQE	IADRGDVIVV	SVGYRVGTLG	166
bsal - Yellow catfish	LQSGTRGSED	CLYLNIWVPQ	GR-SISTGLP	VMVYLFGGGY	LVGGSSGANF	LDNYLYSGEE	IADRGNVIVV	TVNYRVGALG	166
bsal - Channel catfish	IQSGTRGSED	CLYLNIWVPQ	GR-SISTGLP	VMVYFFGGGY	LVGGSQGANF	LDNYLYSGEE	IADRGNVMVV	TVNYRVGALG	166
bsal.1 - Atlantic salmon	LQSDTRGQED	CLYLNIWVPQ	GL-SVSTGLP	VMVWIFGGGY	LVGGSMGANF	LDNYLYDGEE	IANRGKVIVV	TLGYRVGTLG	168
bsal.2 - Atlantic salmon	LQSDTRGQED	CLYLNIWVPQ	GS-SVSTGLP	VMVWIYGGGF	QVGGSMGANF	LDNYLYDGEE	IANRGNVIVV	TLGYRVGTLG	170
bsal.1 - Rainbow trout	LQSDTRGQED	CLFLNIWVPQ	GL-SVSTGLP	VMVWIFGGGY	LVGGSMGANF	LDNYLYDGEE	IANRGKVIVV	TLGYRVGTLG	168
bsal.2 - Rainbow trout	LQSDTRGQED	CLYLNIWVPQ	GS-SVSTGLP	VMVWLYGGGF	QVGGSMGANF	LDNYLYDGEE	IANRGNVIVV	TLGYRVGTLG	170
bsal.1 - Largemouth bass	IMNDVRGSED	CLYLNIWVPH	SG-SVSTDLP	VMVWIYGGGF	LVGGSMGANF	LDNYLYSGQE	IAERGNVIVV	TLGYRVGTLG	190
bsal.2 - Largemouth bass	IMNDVRGSED	CLYLNIWVPH	SG-SVSTDLP	VMVWIYGGGF	LVGGSMGANF	LDNYLYSGQE	IAERGNVIVV	TLGYRVGTLG	190
bsal.3 - Largemouth bass	LQTKTQGSED	CLYLNIFVPQ	G-MKLSTNLP	VMVYLFGGAF	LLGASNEVEI	LGDSLYDGRE	MADRGDVIIV	TVNYRVGTMG	164
bsal.1 - Asian seabass	LQTKTQGSED	CLYLNIFVPQ	G-RSLSTNLP	VMVYLFGGAF	LLGASNDVAI	LGDSLYDGKE	MADRGGVIVI	TVNYRVGTLG	164
bsal.2 - Asian seabass	IMTDTRGSED	CLYLNIWVPH	GR-SVSTGLP	VMVWIYGGGF	LAGGSMGANF	LDNYLYSGQE	IADRGNVIVV	TLGYRVGTLG	167
bsal.3 - Asian seabass	LQTSTFGSED	CLYLNIWVPH	GR-HVSSNLP	VMIWFYGGGF	MVGGSMGPNF	LNNYLYSGQE	IADRGNVIVV	SVGYRVGTLG	166
bsal.1 - Mandarin fish	IMTDTRGSED	CLYLNIWVPH	GR-SVSTDMP	VMVWIYGGGF	LAGGSMGANF	LDNYLYSGQE	IADRGNVIVV	TLGYRVGTLG	167
bsal.2 - Mandarin fish	LQTKTQGSDD	CLYLNIFVPQ	G-RKLSTNLP	VMVYLFGGAF	LLGASNDVAV	LGNSLYDGKE	MADRGGVIVV	TVNYRVGTMG	163
bsal.1 - European seabass	IMTDTRGSED	CLYLNIWVPH	GR-SVSTGLP	VMVWIYGGGF	LAGGSMGANF	LDNYLYSGQE	IADRGDVIVV	TLGYRVGTLG	166
bsal.2 - European seabass	LQTKTQGSED	CLYLNIFVPQ	G-RKLSSNLP	VMVYLFGGAF	LLGASNDVAI	LGDSLYDGKE	MAERGNVIIV	TVNYRVGTLG	170
bsal.1 - Pufferfish	LQTKTRGSED	CLYLNIFVPH	G-GQVSTNLP	VMVYLFGGAF	LLGASNDVAV	LGDSLYDGKE	MADRGGVIVV	TVNYRVGTLG	165
bsal.2 - Pufferfish	PMTDTRGSED	CLYLNIWVPH	GS-SVSTGLP	VMLWIYGGGF	LAGGSMGANF	LDNYLYSGQE	IAERGEVIVV	TVGYRVGTLG	166
bsal.3 - Pufferfish	RMTDTRGSED	CLYLNIWVPH	GS-SVSTDLP	VMLWIYGGGF	MIGGSMGGKF	LDDYLFSGQE	IAERGEVIVV	TVGYRVGTLG	166
bsal.4 - Pufferfish	LQTTSLGSED	CLHLNIWVPQ	GK-HVSSNLP	VMIWIYGGGF	MSGSSTGPGF	LGNYLYSGQE	IADKGDVIVV	SFGYRVGVLG	166
bsal.1 - Japanese flounder	IMTDTRGSED	CLYLNIWVPH	GR-SVSTDLP	VMVWIYGGGF	LAGGSMGANF	LNNYLYSGQE	IADRGNVIVV	TLGYRVGTLG	167
bsal.2 - Japanese flounder	YMTHTRGSED	CLYLNIWVPH	GS-TVSTDLP	VMVWIYGGGF	LTGSSMGSKI	ITTYLYSGQE	MADRGNVIVV	TLGYRVGTLG	166
bsal.3 - Japanese flounder	LQTSSFGSED	CLYLNIWVPH	GR-QVSSNLP	VMIWFYGGGF	MVGGSMGPNF	LNNYLYSGQE	IADKGDVIVV	SVGYRVGTLG	166
bsal.1 - Zebrafish	LATDVIGSED	CLYLNIWVPQ	GR-TVSSNLP	VMVFIYGGAF	LLGGGQGANF	LDNYLYDGEE	MADRGNVIVV	TFNYRVGALG	166
bsal.2 - Zebrafish	LQTDTMGSED	CLYLNIWVPQ	GK-TVSYNLP	VMVFIYGGGF	LLGGGQGANF	LDNYLYDGEE	IADRGNVIVV	TFNYRVGSMG	167
bsal.1 - Medaka	LQTKTHGSEN	CLYLNIFVPQ	G-LSLSTNLP	VMVYLFGGAF	LLGASNDVAI	LGDSLYDGKE	LADRGDVIVV	TVNYRVGTLG	165
bsal.2 - Medaka	TLTNTLGSED	CLYLNIWVPH	GS-SVSTNLP	VMVWIYGGGF	LAGASMGANF	LDNYLYDGQE	LADRGNVIVV	TLGYRVGSLG	166
bsal.3 - Medaka	TMTGTMGSED	CLYLNIWVPH	GS-SVSTGLP	VMVWIYGGGF	VTGGAMGVNF	LNNYLYDGQE	LATRGNVIVV	TLGYRLGPLG	171
bsal.1 - Common carp	LATDVVGSED	CLYLNIWVPQ	GN-TVSTNLP	VMVFIYGGGF	LLGGGQGANF	LDNYLYDGQE	IADRGNVIVV	TFNYRVGSLG	168
bsal.2 - Common carp	LATDVVGSED	CLYLNIWIPQ	GR-SVSTGLP	VMVFIYGGGF	LLGGGQGANF	LNNYLYDGQE	IADRGNVIVV	TFNYRVGSLG	168
bsal.1 - Tilapia nilotica	FQNSYVGSED	CLYLNIWVPH	GS-SVSSGLP	VMVWIYGGGF	MIGGSMGPYY	LDNYMYYGKE	IADRGNVIVV	TLGYRVGPMG	167
bsal.2 - Tilapia nilotica	LMNDYIGSTD	CLYLNIWVPH	GS-SVSAGLP	VMVWIYGGAF	LVGGSMGANF	LDNYLYSGQE	IADRGNVIVV	TVGYRVGTLG	167
bsal.3 - Tilapia nilotica	LMNDYIGSTD	CLYLNIWVPH	GS-SVSAGLP	VMVWIYGGGF	LVGGSMGANF	LDNYLYSGQE	IADRGNVIVV	TVGYRVGTLG	167
Consensus	LQTDTRGSED	CLYLNIWVPQ	GR-SVSTGLP	VMVWIYGGGF	LVGGSMGANF	LDNYLYSGQE	IADRGNVIVV	TVGYRVGTLG	

```
                                                     260                           280                           300                           320
                                                       |                             |                             |                             |
BSAL - Human                  FLSTGDANLP GNYGLRDQHM AIAWVKRNIA AFGGDPNNIT LFGESAGGAS VSLQTLSPYN KGLIRRAISQ SGVALSPWVI 249
Bsal - Mouse                  FLSTGDANLP GNFGLRDQHM AIAWVKRNIA AFGGDPDNIT IFGESAGAAS VSLQTLSPYN KGLIRRAISQ SGMALSPWAI 249
bsal - Spotted gar            FLSTGDANGP GNYGLRDQHA AIAWVHRNIK AFGGDPDNIT IFGESAGSAS VNFQILSPYN KGLIRRAISQ SGMALSPWAL 246
bsal.1 - European eel         FLSSGDASGP GNYGLWDQHA GIAWVHRNIR AFGGDPDNIT VFGESAGAAS VNFQLLSPKN RGLIRRGISQ SGVALSPWAV 246
bsal.2 - European eel         FLSSGDASGP GNYGLWDQHA GIAWVHRNIR AFGGDPDNIT VFGESAGAAS VSFQLLSPKN RGLIRRGISQ SGVALSPWAV 246
bsal.1 - Northern pike        FLSTGDSNAP GNYGLWDQHA AIAWVHRNIQ AFGGAKENIT VFGQSAGAAS VNFQMLSPYN KGLFRWAISQ CGTALSPWAL 320
bsal.2 - Northern pike        FLSSGDVSGP GNYGLWDQHA AIAWVHRNIR AFGGDPNNIT VFGESAGAAS VSFQTLSPHN KGLIRRAISQ SGVALCPWGV 288
bsal.1 - Atlantic cod         FLSTGDARMP GNYGLWDQHA AISWVKRNIE AFGGNPDNIT IFGQSAGAAS VNYQMLSPYS KGLFRRAISQ CGVALSPWAI 247
bsal.2 - Atlantic cod         YLSTGDSSLP GNYGMWDQQA AIAWVHRNIR SFGGDPDNVT LFGESAGGAS VSLQTLSPHN KGLFKRAISQ SGVALCPWAV 248
bsal.3 - Atlantic cod         FLSTGDSGMP GNYGLWDQQA AIAWVSRNIR SFGGDPDNIT VFGESAGSAS VSFQTITPHN KGLFKRAISQ SGTALCPWAV 247
bsal.4 - Atlantic cod         FLSTGDSGMP GNYGLWDQQA AIAWVSRNIR SFGGDPDNIT VFGESAGGAS VSFQTITPHN KGLFKRAISQ SGTAFCPWAV 247
bsal.5 - Atlantic cod         FLSSGDSQLP GNYGLWDQHA AIAWVHRNIR SFGGDPDNVT LFGESAGGSS VSYQTLSPYN KGLFKRAISQ SGVAMCPWAI 246
bsal - Yellow catfish         FLSTGDSELP GNYGLWDQHA AIAWVHRNIK AFGGDPNNIT VFGESAGAAS VSFQMLTPHN KGLIRRAISQ SGVALCPWAV 246
bsal - Channel catfish        FLSTGDSDLP GNYGLWDQQA AIAWVHRNIK AFGGDPNNIT IFGESAGAAS VSFQMLTPHN KGLIRRAISQ SGVALCPWAV 246
bsal.1 - Atlantic salmon      FLSSGDASGP GNYGLWDQHA AIAWVNRNIR AFGGDPNNIT VFGESAGAAS VSFQTLSPHN KGLIRRAISQ SGVALCPWAI 248
bsal.2 - Atlantic salmon      FLSSGDASGP GNYGLWDQHA AIAWVNRNIR AFGGDPNNLT IFGESAGAVS SSFQTLSPHN KGLIRRAISQ SGVALCPWAI 250
bsal.1 - Rainbow trout        FLSSGDASGP GNYGLWDQHA AIAWVNRNIR AFGGDPNNIT VFGESAGAAS VSFQTLSPHN KGLIRRAISQ SGVALCPWAI 248
bsal.2 - Rainbow trout        FLSSGDASGP GNYGLWDQHA AIAWVNRNIR AFGGDPNNLT IFGESAGAVS SSFQTLSPHN KGLIRRAISQ SGVALCPWAI 250
bsal.1 - Largemouth bass      FLSTGDSSLP GNYGMWDQQH AIAWVHRNIR SFGGDPDNIT IFGESAGAAS VSMQTLTPHN KGLFKRAISQ SGVALCPWAI 270
bsal.2 - Largemouth bass      FLSTGDSSLP GNYGMWDQQH AIAWVHRNIR SFGGDPDNIT IFGESAGAAS VSMQTLTPHN KGLFKRAISQ SGVALCPWAI 270
bsal.3 - Largemouth bass      FLSTGDARLP GNYGLWDQHA AISWVRRNIQ AFGGNPDNIT IFGQSAGAAS VSYQMLSPYS KGLFRRAITQ SGVALSPWAL 244
bsal.1 - Asian seabass        FLSTGDARLP GNYGLWDQHA AISWVRRNIE AFGGNPDNMT IFGQSAGAAS VSYQMLSPYN KGLFRRAITQ CGVALSPWAL 244
bsal.2 - Asian seabass        FLSTGDSSLP GNYGLWDQHT AIAWVHRNIR SFGGDPDNIT IFGESAGGAS VSFQTLTPHN RGLIKRAISQ SGVALCPWGV 247
bsal.3 - Asian seabass        FLSTGDSTLP GNYGLWDQHA AIAWVHRNIR SFGGDPENIT LFGESAGGAS VSFQTLTPHN KGLFKRAISQ SGVAFCPWAF 246
bsal.1 - Mandarin fish        FLSTGDSSLP GNYGLWDQHA AIAWVHRNIR SFGGDPDKIT VFGESAGGAS VSFQTLTPHN KGLFKRAISQ SGVALCPWAV 247
bsal.2 - Mandarin fish        FLSTGDGRLP GNYGLWDQHA AISWVRRNIR AFGGNPDNVT IFGQSAGAAS VSFQMLSPYS KGLFRRAISQ GGVALSPWAL 243
bsal.1 - European seabass     FLSTGDSGLP GNYGLWDQHT AIAWVHRNIR SFGGDPDNII IFGESAGGAS VSFQTLSPHN KGLIKRAITQ SGVALCPWAL 246
bsal.2 - European seabass     FLSSGDARLP GNYGLWDQHA AISWVRRNIE AFGGNPDNIT IFGQSAGAAS VSFQMLSPYS KGLFRRAISQ CGVALSPWAL 250
bsal.1 - Pufferfish           FLSTGDARLP GNYGLWDQHA AISWVRRNIR AFGGNPDNIT IFGQSAGAAS VSYQMLSPYS RGLFRRAISQ CGVALSPWAL 245
bsal.2 - Pufferfish           FLSTGDSSLP GNYGLWDQQA AIAWVHRNIR SFGGDPDNIT LFGESAGGAS VSFQTLTPHN KGTIRRAISQ SGVALCPWAV 246
bsal.3 - Pufferfish           FLSTGDSSLP GNYGLWDQQA AIAWVHRNIR SFGGDPDNIT LFGESAGGAS VSFQTLTPHN KGTIRRAISQ SGVALCPWAV 246
bsal.4 - Pufferfish           FLSTGDSRLP GNYGLWDQHA AIAWVHRNIR LFGGDPDNIT IFGESAGGAS VSFQTLSPHN KGLVRRAISQ SGDALSPWAV 246
bsal.1 - Japanese flounder    FLSTGDSSLP GNYGLWDQQA AIAWVHRNIR SFGGDPDNVT VFGESAGAAS VSFQTLTPHN KGLFKRAISQ SGVALCPWAI 247
bsal.2 - Japanese flounder    FLSTGDSSLP GNYGLWDQQA AIAWVHRNIR SFGGDPDS-- ---------- ---------- ---------- ---------- 204
bsal.3 - Japanese flounder    FLSTGDSNLP GNYGLWDQHA AIAWVHRNIR SFGGDPDNVT IFGESAGGAS VNFQMLSPHN KGLFNRAISQ SGVALCPWAL 246
bsal.1 - Zebrafish            FMSTGDDGIP GNYGLWDQHA AISWVHRNIK AFGGNPDNIT LFGESAGAAS VNFQIITPKN KGMIRRAISQ SGVALCPWAI 246
bsal.2 - Zebrafish            FLSTGDADAP GNYGLWDQHA AISWVHRNIK AFGGNPDNIT IFGESAGSTS VNFQIISPKN KGLIRRAISQ SGVALCPWAI 247
bsal.1 - Medaka               FLSTGDDRLP GNYGLWDQHA AISWIRRNIA AFGGHPDNLT IFGQSAGAAS VSYQMISPYS KGLFRRAISQ CGVALSPWAL 245
bsal.2 - Medaka               FLSTGDSELP GNYGLWDQQA AIAWVHRNIR AFGGDPSNIT VFGESAGGAS VNFQILTPHN KGLIRRAISQ SGVALCPWAV 246
bsal.3 - Medaka               FLSTGDSDIP GNYGLWDQQA AIAWVHRNIG SFGGDPSNIT IFGESAGGAS VSLQTLTPHN KGIIRRAISQ SGVAMCPWVI 251
bsal.1 - Common carp          FLSSGNAEAP GNYGLWDQHT AIAWVHRNIR NFGGNPGNIT IFGESAGAAS VNFQILSPKN KGLIRRAISQ SGVALCPWAV 248
bsal.2 - Common carp          FLSSGDADAP GNYGLWDQHA AISWVHRNIR NFGGNPDNIT IFGESAGGAS VNFQILSPKN KGLIRRAISQ SGVALCPWAV 248
bsal.1 - Tilapia nilotica     FMSTGDSDLP GNYGLWDQQA AIAWVHRNIR SFGGDPDNIT IFGESAGGVS VSFQTLTPHN KGIIRRAISQ SGVALCPWAL 247
bsal.2 - Tilapia nilotica     FLSSGGSDLP GNYGLWDQQA AIAWVHRNIR SFGGDPGNIT VFGESAGGAS VSFQTLTPHN KGIIRRAISQ SGVALCPWGI 247
bsal.3 - Tilapia nilotica     FLSSGDSGLP GNYGLWDQQA AIAWVHRNIR SFGGDPGNIT VFGESAGGAS VSFQTLTPHN KGIIRRAISQ SGVALCPWGI 247
Consensus                     FLSTGDSSLP GNYGLWDQHA AIAWVHRNIR AFGGDPDNIT IFGESAGAAS VSFQTLSPHN KGLIRRAISQ SGVALCPWAX
```

```
                                    340                  360                  380                  400
                                      |                    |                    |                    |
BSAL - Human              QKNPLFWAKK VAEKVGCPVG DAARMAQCLK VTDPRALTLA YKVP--LAGL -EYPMLHYVG FVPVIDGDFI PADPINLYAN 326
Bsal - Mouse              QKNPLFWAKT IAKKVGCPTE DTGKMAACLK ITDPRALTLA YKLP--VKKQ -EYPVVHYLA FIPVIDGDFI PDDPINLYNN 326
bsal - Spotted gar        NRNALYWAKK IAEKVGCPTD DTARLMGCLK ITDPVAVTLA GDLM--LFGS SD-PVVWNLG LSPVVDGDFI PDDPGNLFHN 323
bsal.1 - European eel     NRNPRALAEE IAIKVGCPTD E--KMMPCLK ITDPVALTLA GSLH--LKGS PSNPLLFNLL LAPVIDGDFL PDEPGNLFHN 322
bsal.2 - European eel     NRNPRALAEE IAIKVGCPTD E--RMMPCLK ITDPVALTLA GTQN--LDGS PSNPIVFNLL LTPVIDGDFL PDEPGNLFHN 322
bsal.1 - Northern pike    QTNPLPLANK IAEKVGCATD --EQMMTCLK ITEPVALTLA RKIE-LGELG K-GSVLELLE HAPVVDGDFV PEHPSKLFHN 396
bsal.2 - Northern pike    NKNPRAFAEM VAEKVGCPKD D--QMMTCLK LTDARDLTLA GTLQ--LNGS PSTPIVDNLA LSPVIDGDFL PDHPGKLFHN 364
bsal.1 - Atlantic cod     QLDPMATTKK VARKVGCRTT NEEEMLLCLK ITDPVGITMA AKLNLLLMLG P-SPVMNLLE FTPVVDGDFI PDHPSRLFNN 326
bsal.2 - Atlantic cod     NRNPRPVAES VARKVNCPTD E--TMASCLK MTDPVALTMA GNMP--YTSS PDSPLLNNLV LAASIDGDFL PDDPGNLFNN 324
bsal.3 - Atlantic cod     NRNPRRLAEE VALKVNCPTD D--RMAACLK MTDPVSLTMA GTIP--ITSS PDSPLVFNLV LAATIDGDFL PDEPSTLFHN 323
bsal.4 - Atlantic cod     NRNPRRSAEE VALKVNCPTD D--RMAACLK MTDPVSLTMA GTIS--FSSS PDSPLVFNLV LAATIDGDFL PDEPSTLFHN 323
bsal.5 - Atlantic cod     SRNPRNVAEE VAVKVGCPTD D--KMVACLK SVNAKKLVMA APLF--VSGS PDKPALNAMV LSPVVDGDFL PEDPVRLFHN 322
bsal - Yellow catfish     NRNPRAFAEE VAKKVGCPTD Q--SMAACLK MTDPVELTAA GTLN--LKGS ATNPIVKNLA LAPVIDGDFL PDDPSTLFGN 322
bsal - Channel catfish    NRNPRAYAEE VAKKVGCPTD Q--SMAACLK MTDPVKLTLA GTLN--LKGS ATNPIVKNLA LSPVIDGDFL PAAPSTLFSN 322
bsal.1 - Atlantic salmon  NHNPRAFAEM VAGKVGCPTD D--QMMACLK LINAKELTLA GTLS--LAGS PSTPIVDNLA LSPVIDGDFL PDHPGNLFHN 324
bsal.2 - Atlantic salmon  NMNPRAFAEK VAEKVGCPKD D--QMMACLK LIDAKVLTLA GTTS--MSGS PSTPMVDNLV LSPVIDGDFL PDHPGNLFHN 326
bsal.1 - Rainbow trout    NNNPRAFAEM VAGKVGCPID D--HMMACLK LIDAKELTLA GTLS--LAGS PSTPIVGNLA LSPVIDGDFL PDHPGKLFHN 324
bsal.2 - Rainbow trout    NRNPRAFAEN VAEKVGCPKD D--QMMACLK LVDANVLTLA GTTA--LGGS PSTPMVDNLV LSPVIDGDFL PDHPGNLFHN 326
bsal.1 - Largemouth bass  NKNPRRFAEE VALKVNCPTD Q--TMAACLK MTDPVLLTMA GTLS--LSSS ADHPLVFNLA LSPVIDGDFL PDEPYNLFHN 346
bsal.2 - Largemouth bass  NKNPRRFAEE VALKVNCPTD Q--TMAACLK MTDPVQLTMA GTLS--LSSS ADHPLVFNLA LSPVIDGDFL PDEPYNLFHN 346
bsal.3 - Largemouth bass  QKNPLALTKK IARKVGCWRS NVDEMVACLK ISDPVGLTMA GKIDVLLILG K-GVVMDLLE FSPVVDGDFI PDEPSKLFHN 323
bsal.1 - Asian seabass    QKKPMALTKK IARKVGCSRT DVDEMITCLK MSDPVGLTMA GKIDVLLILG K-GVVMDLLE FAPVVDGDFI PDEPSKLFHN 323
bsal.2 - Asian seabass    IKNPRRIAEE VALKVNCPTD D--KMAACLK MTDPVLLTMA GTLS--LSSS PDNPLVNNLV LSAVIDGDFL PDEPTNLFHN 323
bsal.3 - Asian seabass    SRNPRKVAEE VAVKVGCPTD D--RMVACLK STDAGTLTMA SPRI--QQGS PDYPGVKNLL LSPVVDGDFL PDQPENLFHN 322
bsal.1 - Mandarin fish    NKNPRRFAEE VALKVNCPTD Q--TMAACLK MTDPALLTLA GSLS--LSSS PDHPLVYNLA LSPVIDGDFL PDEPYNLFHN 323
bsal.2 - Mandarin fish    QKNPMALTKK IARKVGCSRK DVDVMLTCLK MSDPVGLTLA GKINVLLLLG K-GVVMDLLQ LAPVIDGDFI PDEPSQLFHN 322
bsal.1 - European seabass YKNPQQVAEE VAVKVGCPTD D--RMVACLK STDAVTLTMA VPSR--PQGS PDHPGVENML LSAVVDGDFL PDQPANLFHN 322
bsal.2 - European seabass QNNPMALTKK VARRVGCWRT NIDLMITCLK MSDPVGLTMA GKIDVLLILG K-GVVMDLLE FSPVVDGDFI PDEPSKLFHN 329
bsal.1 - Pufferfish       QKNPMALTKK IARKVGCWRS NEDEMLTCLK MSDPSGLTMA GKIDVLLLLG K-GVVMDLLE LAPVVDGDFI PDNPSRLFHN 324
bsal.2 - Pufferfish       NRNPRRFAEE VALKVNCPTD E--KMAACLK MTDPELLTLA GSLK--MSGS PDNPLVSNLV LSPVIDGDFL PDEPYNLFHN 322
bsal.3 - Pufferfish       NRNPRRFAEE VALKVNCPTD E--KMAACLK MTDPELLTLA GSLN--KSRS PDNPLVSNLV LSPVIDGDFL PDEPYNLFHN 322
bsal.4 - Pufferfish       NEDPRTMAER VALKAGCPVD E--RMVACLR SSDARNLTMS TPYI--PDGT PDYPGIKHVL LTPVVDGDFL PDEPVNLFHN 322
bsal.1 - Japanese flounder NKNPRKFAEE VALKVNCPTG E--NMAACLK MTDPALLTLA GSLS--LSSS PDSPVVGNLL LSPVIDGDFL PDAPHNLFHN 323
bsal.2 - Japanese flounder ---------- ---------- ---------- ---------- ---------- ---PIVENLL LSPVIDGDFL PDAPHNLFHN 231
bsal.3 - Japanese flounder NRNPRKITEE VAVKVGCPTD D--RMVECLK STDAATLCMA SPLI--QPGS PDHPGVENLL LSPVVDGDFL PDQPSNLFQN 322
bsal.1 - Zebrafish        SRNPRQFAEE IATKVGCPID S--GMADCLK RADPKAVTLA GKLK--LTSS PDAPIVHNLY LSPVIDGDFI PDEPETLFGN 322
bsal.2 - Zebrafish        NRNPRKFAEE IAKKVGCPTD S--GMVACLR RTDPKAVTLA GKVR--LATS ATEPIVHNLY LSPVIDGDFI PDEPDTLFGN 323
bsal.1 - Medaka           QRNPLKVTKK IARKVGCSNT NVDEMIACLK GSNPVDLTLA GKIDVLLILG KAGVVMDLLQ LAPVIDGDFI PNEPSELFHN 325
bsal.2 - Medaka           NHNPRKFAEE VALRVNCPTD S--RMAACLK MTDPITLTKA GTIH--LSGS PDQPIVNNLI LSPVIDGDFL PDEPSNLFHN 322
bsal.3 - Medaka           SKHPRKFAEE VALKVNCPID S--TMAACLK TIDSATLTVA GTIN--MSGS PDNPVVNNLV LSPVVDGDFL PDEPHNLYHN 327
bsal.1 - Common carp      NRNPRQYAEE IARKVGCPTD S--GMVACLR RTDPKAVTLA GNVK--FSTS ASHPIVHNLA LSPVIDGDFL PAEPDTLFGN 324
bsal.2 - Common carp      NRNPRQYAEE IARKVGCPTD S--GMVACLR RTDPKTVTLA GNVN--FSAS ASHPIVHNLA LSPVIDGDFL PDEPETLFGN 324
bsal.1 - Tilapia nilotica NRNPRRFAEE VARKVNCPTD S--RMASCLK MTDPGTLTKA GTVN--LLSS PDQPLVLNLE IAPVIDGDFL PDDPSNLFHN 323
bsal.2 - Tilapia nilotica NRNPRKFAEE VAQKVNCPTD N--RMAACLK MADPGALTLA GPLD--VSAS PDNPIVFNLV LSPVIDGDFL PDDPSHLFHN 323
bsal.3 - Tilapia nilotica NRNPRKFAEE VAQKVNCPTD N--RMAACLK MTDPGALTLA GTIS--LSGS PDNPIVFNLV LSPVIDGDFL PDDPSHLFHN 323
Consensus                 NRNPRAFAEE VALKVGCPTD D--RMAACLK MTDPVALTLA GTLS--LSGS PDNPIVDNLV LSPVIDGDFL PDEPSNLFHN
```

		420		440		460		480	
BSAL - Human	AADIDYIAGT	NNMDGHIFAS	IDMPAINKGN	KKVTEEDFYK	LVSEFTITKG	LRGAKTTFDV	YTESWAQDPS	QENKKKTVVD	406
Bsal - Mouse	TADIDYIAGI	NNMDGHLFAT	IDVPAVDKTK	QTVTEEDFYR	LVSGHTVAKG	LKGAQATFDI	YTESWAQDPS	QENMKKTVVA	406
bsal - Spotted gar	AADIDYIAGI	NNMDGHLFAG	LDVPSINRAK	ETTNPEDLKR	LLRGLTREKG	EQAVQSAFEL	YTQNWGPSPS	QETIKKTVVD	403
bsal.1 - European eel	AADIDYIVGI	NNMDGHLFCG	IDVPSINQPL	QPTPPEVAME	LLTALTREKG	PEAASLAYKE	YTQSWGSTPS	KSTIKQTIAE	402
bsal.2 - European eel	AADIDYIAGI	NNMDGHLFCG	IDVPSINQPL	QPTPPEVAME	LLNALTKEKG	PEAASLAYKE	YTQSWGSTPS	KSTIKQTITE	402
bsal.1 - Northern pike	AANIDYLAGV	NSMDGHLFAG	VDVPSINKNQ	MPTT------	----------	----------	----------	----------	430
bsal.2 - Northern pike	AADIDYLAGI	NSMDAHLFAG	MDVPDINGVI	-SVPVSDVKL	LLGALTK-KG	ENVTNSALAE	YSADWGTKPS	QETIKKTVVA	442
bsal.1 - Atlantic cod	TADIDYLAGV	NSMDGHLFAG	VDVPSINQRR	KETTAAQVKK	LISGLTQQKG	AEAIQSVYSM	YSANWGSVPQ	QDMVKKTVTD	406
bsal.2 - Atlantic cod	TADIDYLAGV	NNMDGHLFTG	LDIPSINRPL	VDTKVEDLRA	LLGAYTKVKG	KAGQEAAFSQ	YSTGWGANPS	KETVKRTVVD	404
bsal.3 - Atlantic cod	AADIDYIAGA	NDMDGHLFTG	LDVPTINSHL	LHTPVNDVKR	LLGSYTREKG	LAGMESGFLT	YSSTWGTNPD	QETIKKTVVE	403
bsal.4 - Atlantic cod	AADIDYIAGV	NDMDGHFFEG	LDVPTINSHL	LHTPVNDVKR	LLGSYTKEKG	LAGMESGFLT	YSSTWGTNPD	QETIKKTVVE	403
bsal.5 - Atlantic cod	AADIDYLAGV	NSMDGHLFTS	QDIPAI-ADK	GNITVDDVTA	LLRFYTKDKG	QAGLEAALAE	YTAHWGPSPS	QDQVKITAVE	401
bsal - Yellow catfish	AADIDYIAGV	NNMDGHLFTG	FDVPSVNQPL	QPTPVEDVQA	LLTALTSDKG	PEASAAAYNQ	YTASWGSKPG	KDDIKKTIID	402
bsal - Channel catfish	AADIDYIAGV	NDMDGHIFTG	FDIASINQPL	QPTPIEDVKA	LLTALTSDKG	LEASTAAYDQ	YTANWGSKPG	KDDIKKTIVE	402
bsal.1 - Atlantic salmon	AADIDYLAGV	NSMDAHLFTG	LDLPAVNKPI	ANLPLSDVKL	LLGSLTK-KG	EASINSAFAE	YTADWGDKPS	QETIKKTVVM	403
bsal.2 - Atlantic salmon	AADIDYLAGV	NSMDAHLFTG	QDIPNINNPS	ENVPVSDVKL	LLGSLTK-KG	EAATNNAFAE	YTADWGDEPS	QKTIKKTVVM	405
bsal.1 - Rainbow trout	AADIDYLAGV	NSMDAHLFTG	LDLPAVNKPL	ANLPLSDVKL	LLGSLTK-KG	EASINSAFAE	YTADWGDKPS	QETIKKTVVM	403
bsal.2 - Rainbow trout	AADIDYLAGV	NSMDAHLFAG	QDIPNINNPS	ANVPVSDVKL	LLSSLTK-KG	EAATNNAFTE	YTADWGDEPS	QKTIKKTVVM	405
bsal.1 - Largemouth bass	AAEIDYIAGV	NDMDGHLFTG	LDVPSINSPL	VDTPIEDVKR	LLAAYTKEKG	KAGLDNAYST	YTSTWGSNPS	KETVKKTVVE	426
bsal.2 - Largemouth bass	AAEIDYIAGV	NDMDGHLFTG	LDVPSINSPL	VDTPIEDVKR	LLAAYTKEKG	KAGLDNAYST	YTSTWGSNPS	KETVKKTVVE	426
bsal.3 - Largemouth bass	AAQFDYLAGV	NSMDGHFFAG	VDVPNINIKN	V-TTVKQVKG	LLAGLTKEKG	KAAIESAYSV	YSSNWGVAPE	QAVVKKTVAD	402
bsal.1 - Asian seabass	AAQFDYMAGV	NSMDGHIFAG	VDVPSINQKN	GNTTVDDVKG	LLVGLTKEKG	NAAVSSAYSV	YSAHWGSAPE	PAVVKKTVAD	403
bsal.2 - Asian seabass	AADIDYIAGV	NDMDGHIFTG	LDIPSINAPL	VDTPIEDMKR	LLASYTKDKG	KAGLDNAYST	YTSSWESNPS	KETVKRTIVD	403
bsal.3 - Asian seabass	TADIDYLVGV	NDSDGHLFTS	QDIPSLGNKN	EETPVEDVKR	LLAAYTKEKG	QAGLEIAFAE	YSSNWGVNPS	QDTIKKTAVD	402
bsal.1 - Mandarin fish	AADIDYIAGV	NDMDGHLFTG	LDVPSINSPL	VDTAIEDMKR	LLAAYTKEKG	KAGLDNAYST	YTSTWGSNPS	REMVKKTVVE	403
bsal.2 - Mandarin fish	TAHFDYLAGV	NSMDGHFFAG	VDVPSINKKN	V-TSVEQVEG	LLAGLTKEKG	NAAVSSAFGA	YSSHWGSAPE	QEVVKKTVVD	401
bsal.1 - European seabass	AADIDYLAGV	NDMDGHVFTS	MDIPSLGNKN	EETPVEDVKR	LLAAYTKEKG	QAGLEIAFAE	YSSDWGSAPS	QNTIKRTAVD	402
bsal.2 - European seabass	AAQFDYLAGV	NSMDGHFFAG	VDVPNINQKN	A-TTVAQVRG	LLAGLTKEKG	NAAIDSAYST	YTSHWGSAPE	QAVVKKTVAD	408
bsal.1 - Pufferfish	AARFDYMAGV	NSMDGHFFAG	VDVPNINTRN	-ETTVSQVRG	LLAGLTKEKG	SAAVDSAFGV	YSAHWGSAPE	QAMVKKTVAD	403
bsal.2 - Pufferfish	AADIDYIAGV	NDMDGHLFTA	FDIPSINSQL	VDTPVDEMKR	LLRSYTKEKG	AEAAEIGFST	YTLSWGSNPN	RETIKTTVVD	402
bsal.3 - Pufferfish	AADIDYIAGV	NDMDGHLFTT	FDIPSINSQL	VDTPVDEMKR	LLRSYTKEKG	AEAAEIGFST	YTLNWGSNPN	RETIKKTVVD	402
bsal.4 - Pufferfish	TAEIDYLAGV	NDMDGFSFTS	EDIHSLKNKT	EKTPVDEVIR	LLAAYTKDTG	PEGFQVASDE	YFSKWGASPS	QETVQRTAVE	402
bsal.1 - Japanese flounder	AADIDYIAGI	NDMDGHLFTG	LDVPSINSPL	VDTSVDDVKR	LLASYTKDKG	KAGFDNAYST	YTSTWGSNPS	RETIKRTVVE	403
bsal.2 - Japanese flounder	AADIDYIAGI	NDMDGHLFTS	LDVPSINLPL	VGTSVDVVKR	LLASYTKDKG	QAGSDIAFST	YNSTWGSNPS	RETIKRTVVE	311
bsal.3 - Japanese flounder	AADIDYLAGV	NNMDGHLFTS	NDIPSLGNKN	QDTPVEDVRR	LLAAYTKEKG	QAGLDVAFSE	YSSNWGSDPS	QETIKRTAVD	402
bsal.1 - Zebrafish	AADIDYIAGV	NDMDAHIFAT	IDIPSINNAL	TTTPVEEVQA	LATALSRDRG	QDAGIATFQE	YTVNWGDKPN	KEKVKQTVVE	402
bsal.2 - Zebrafish	AADIDYIAGV	NDMDGHIFAT	LDIPSINNAL	ATTTEEEVQA	LATALSKDRG	QDAGIATFQE	YTVNWGSKPN	KEDIKKTVVE	403
bsal.1 - Medaka	AAQFDYLAGI	NSMDGHFFAG	VDVPSINLGN	ANTTADHVKG	LLAGLTKEKG	DAAIPSAFSV	YSTHWGSFPP	PDVVKKTATD	405
bsal.2 - Medaka	AAEVDYIAGA	NDMDGHIFTG	LDVPSVNNDI	VETPIEDVKR	LLGALTKEKG	QLGLENAYST	YSSDWGSNPS	WQKVKQTVVA	402
bsal.3 - Medaka	AAEIDYLAGT	NDMDGRMYSS	MDVPSVNDDV	LETSIEDVKR	LLTALTKEKG	QMGLDGAYAT	YSASWGSSPS	QETVKKTVVA	407
bsal.1 - Common carp	AADIDYIAGV	NDMDGHIFAT	LDIPSINNAL	VSTPVEEVKK	LSIALSESRG	TDAGIATYEE	YTANWGSKPS	DTDIKKTVVD	404
bsal.2 - Common carp	AADIDYIAGV	NDMDGHIFAT	LDIPSINNAL	VSTPVEEVRA	LSVALSKSRG	PDAGITTYEE	YTVNWGSKPS	DKVVKKTIVD	404
bsal.1 - Tilapia nilotica	AAEIDYIAGV	NNMDGHMFTN	FDVPSVNLDL	VVTPTNDVRR	LLAALTKEKG	ESGLNNAYST	YTSNWGSFPT	WDTIKKTIVD	403
bsal.2 - Tilapia nilotica	AAEIDYIAGV	NDMDGHIFTA	LDVPSINSDL	VDTPIDDVRR	LLGAYTKEKG	AVGLNNAYST	YTSNWGSNPS	QETIKKTIVG	403
bsal.3 - Tilapia nilotica	AAEIDYIAGV	NDMDGHIFTA	LDVPSINSDL	VDTPIDDVRR	LLGAYTKEKG	AVGLNNAYST	YTSNWGSNPS	QETIKKTIVG	403
Consensus	AADIDYIAGV	NDMDGHLFTG	LDVPSINNPL	VDTPVEDVKR	LLAALTKEKG	QAGLXSAFSX	YTSNWGSNPS	QETIKKTVVD	

```
                                                    500                     520                     540                     560
                                                      |                       |                       |                       |
BSAL - Human                FETDVLFLVP TEIALAQHRA NAK---SAKT YAYLFSHPSR MP----VYPK WVGADHADDI QYVFGKPFAT PTGYRPQDRT 479
Bsal - Mouse                FETDVLFLIP TEIALAQHKA HAK---SAKT YSYLFSHPSR MP----IYPK WMGADHADDL QYVFGKPFAT PLGYRPQDRA 479
bsal - Spotted gar          VETDFLFLAP TESGLYLHAN NSK---TGRT YFYVFSMPSR IP----IFPK WMGADHAEDL QYVFGKPFTT PLAYFPRHRD 476
bsal.1 - European eel       IETDFIFLVP TQATLYQHAN MSK---TGRT YSYQFSVPSR ----IPFYPS WMGADHAEDL QYVFGKPFTT PLAYFPRHRT 475
bsal.2 - European eel       IETDFIFLVP TQAALYQHAN MSK---TGRT YSYQFSVPSL ----ITTYPG WMGADHADDL QYVFGKPFTT PLAYSPRHRD 475
bsal.1 - Northern pike      ---------- ---------- --S---GART YSYLFNMGNR IPG----FPS WVEAEHEEDV QYVFGKPFDT PLGYFPRHRT 481
bsal.2 - Northern pike      IETDYIFLVP TQVSLYLHAS NAQ---SAQT YSYVFSEPSR LAGAVKPYPS WMEADHAADL QYVFGKPFTT PLAYWPRHRD 519
bsal.1 - Atlantic cod       IETDYLFLVP TQVALQLHAQ HAR---GART YSYLFDMKTR IPG----LPS WVGAMHAEDV QYLFGKPFDK PLIYFPRHRD 479
bsal.2 - Atlantic cod       IGTDYIFLIP TQSALYLHAS HAK---SGRT YSYLFSEPNR LGGLAKPYPS WMGADHTDDL QFVFGKPFSS WLGYWPSHRN 481
bsal.3 - Atlantic cod       IGTDYIFLIP TQAALYLHA- NATIGRSGRT YSYLFSQPNR MGGLVLPYPS WMGADHADDL QYVFGKPLTS PLGYWPSHRD 482
bsal.4 - Atlantic cod       IGTDYIFLIP TQAALYLHA- NATIGRSGRT YSYLFSQPNR MGGLVLPYPS WMGADHADDL QYVFGKPLSS PLGYWPSHRD 482
bsal.5 - Atlantic cod       VGTDHYFLAP VQTAIYLHAA AAK---SGHT YSYLLSEPSL LIGPGRPYHT WVGADHADDL QYVFGKPFTT PKAYGDAQRD 478
bsal - Yellow catfish       IETDYTFLVP TQTSLYLHAK HAK---TGRT YSYVFSEPSR LPG----YPT WMGADHADDL QYVFGKPFTT PLAYLPKHRR 475
bsal - Channel catfish      IETDYTFLVP TQTALYLHAK HAK---TGRT YSYLFSEPSR MPF----YPS WMGADHADDL QYVFGKPFTT PLGYFPRHRR 475
bsal.1 - Atlantic salmon    IETDYVFLVP TQAALYLHAS NAQ---SART YSYLFSEPSR MSGVVLPFPS WMEADHAEDL QFVFGKPFTT PLAYWPKHRN 480
bsal.2 - Atlantic salmon    IETDYIFLVP TQVALYLHAS NAQ---SVRT YSYLFSEPSR MAGIVQPYPS WMEADHADDL QYVFGKPFTT PLAYWPSHRD 482
bsal.1 - Rainbow trout      IETDYVFLVP TQAALYLHAS NAQ---SART YSYLFSEPSR MSGIVLPFPS WMEADHAEDL QFVFGKPFST PLAYWPKYRD 480
bsal.2 - Rainbow trout      IETDYIFLVP TQVALYLHAS NAQ---SART YSYLFSEPSR MAGIVQPYPS WMEADHADDL QYVFGKPFTT PLAYWPSHRD 482
bsal.1 - Largemouth bass    IGTDYIFLVP TQTALYLHAA NAT---TGRT YSYLFSQPNR MGGIGRPYPS WMGADHADDL QYMFGKPFTT PLGYWPRHRD 503
bsal.2 - Largemouth bass    IGTDYIFLVP TQTALYLHAA NAT---TGRT YSYLFSQPNR MGGIGRPYPS WMGADHADDL QYMFGKPFTT PLGYWPRHRD 503
bsal.3 - Largemouth bass    IETDFLFLVP TQIALQLHAD NSS---GART YSYLFNMKTR IPG----FPR WVEAEHAEDL QYLFGKPFTY ALLYFPRHRD 475
bsal.1 - Asian seabass      IETDFLFLVP TQIALQLHAN NSS---GART YSYLFNMKTR IPG----FPR WVEAEHAEDL QYLFGKPFST PLVYFPRHRD 476
bsal.2 - Asian seabass      IGTDYIFLVP TQAALYLHAA NAT---TART YSYLFSQPNR MGGIGRPYPS WMGADHADDL QYVFGKPFTT PLAYWPRHRD 480
bsal.3 - Asian seabass      IGTDYIFLVP IQAAIYLHAA NAR---SGRT YSYLLSEPSL MAGPGKPFHD WVGSDHADDL QFVFGKPFTT PKAYGDRHRD 479
bsal.1 - Mandarin fish      IGTDYIFLVP TQTALYLHAA NAT---TGRT YSYFFSQPSR MGGIGRPYPS WMGADHADDL QYVFGKPFTT PLGYWPSHRS 480
bsal.2 - Mandarin fish      IETDFLFLVP TQIALQLHAN NSS---GART YSYLFDMKTR IPG----FPH WVEAEHAEDL QYLFGKPFSI PLVYFPRHRD 474
bsal.1 - European seabass   IGTDYMFLVP AQTTIYLHAA NAR---SGRT YSYLLSEPSL LAGPGKPYHD WVGADHADDL QYVFGKPFTT PKAYGDRHRD 479
bsal.2 - European seabass   IETDFLFLVP TQIALQLHAN NSS---GAHT YSYLFNMKTR IPG----FPH WVEAEHAEDL QYVFGKPFAT PLVYFPRHRD 481
bsal.1 - Pufferfish         IETDFLFLVP TQLALQLHAN NSS---GART YSYLFNMKTR IPG----FPA WVEAEHAEDL QYLFGKPFSM PLVYFPRHRD 476
bsal.2 - Pufferfish         VGTDYIFLVA TQAALYLHAA HAK---TRRT YSYMFSEPNR LGGITKPYPS WMGADHADDL QYVFGKPFTT PLGYWPRHRD 479
bsal.3 - Pufferfish         VGTDYIFLVA TQAALYLHAA HAK---TRRT YSYMFSEPSR LGGIAKPYPS WMGADHADDL FYVFGKPFTT PIIYWPRHRD 479
bsal.4 - Pufferfish         IGTDYIFLVS TQAALYLHAA KAT---SAGT FSYLLSEPSL LAGPGRPYPD WMGADHTDDL QYVFGIPFAI PQVYGDKQRE 479
bsal.1 - Japanese flounder  IGTDYIFLVP TQAALYLHAA NAT---TGRT YSYLFSQPNR MGGIGRPYPS WMGADHADDL QYMFGKPFTT PLAYWPRHRD 480
bsal.2 - Japanese flounder  IGTDYIFLVP TQAALYLHAA NAT---TGRT YSYLFSQPNR MGGIGRPYPS WMGADHADDL QYMFGKPFTT PLAYWPRHRD 388
bsal.3 - Japanese flounder  IGTDYIFLVA IQTAIYLHVA NAR---SGRT FSYLLSEPSL MAGPGKPYND WVGADHADDL QFVFGKPFTT PSAYGDRHRD 479
bsal.1 - Zebrafish          LETDYMFLVP TQAALYLHTD NAK---SART FSYLFTESSR IPV----FPL WMGADHADEL QYVFGKPFAT PLGYFPRHRD 475
bsal.2 - Zebrafish          METDYIFLVP TQTALYLHSD NAK---SART YSYLFSESSR IPI----FPL WMGADHADEL QYVFGKPFTT PLGYFPRHRD 476
bsal.1 - Medaka             IETDFLFLVP TQAALQLHTN YSS---GART YSYLFNMKTR IPG----FPW WVEANHAEDL QYLFGKPFST PLGYAPRHRD 478
bsal.2 - Medaka             IETDYIFLIP TQTALYLHAE KAT---TGRT YSYLFSEPNR MGGLIAPFPI WMGADHADDL QYVFGKPFST PLGYWPRHRD 479
bsal.3 - Medaka             IETDYLFLIP TQTALYLHAG SAT---SGRT YSYLFSVPNR MGGIMPSFPS WMGADHTDDL QYVFGKPLST PLIYRAEDKA 484
bsal.1 - Common carp        METDYIFLVP TQATLYLHSD HAK---SGRT YSYLFNEPSR IPV----FPL WMGADHADDL QYVFGKPFTT PLGYFPRHRD 477
bsal.2 - Common carp        METDYIFLVP TQAALYLHSD HAK---SGRT YSYLFSEPSR MPV----FPV WMGADHADDL QYVFGKPLST PLGYFPRHRD 477
bsal.1 - Tilapia nilotica   IETDYIFLVP TQAALYLHAD HAT---TGRT YSYLFSEPNL LGGPLLPYPS WMGADHADDL QYVFGKPFSS PLLYWPSHRR 480
bsal.2 - Tilapia nilotica   VGTDYIFLVP TQAALYLHAD HAT---TGRT YSYLFSEPNR LGGPLLPYPS WMGADHADDL QYVFGKPFST PLGYWDNHRR 480
bsal.3 - Tilapia nilotica   VGTDYIFLVP TQAALYLHAD HAT---TGRT YSYLFSEPNR MGGLIMPYPS WMGADHADDL QYVFGKPFTT PLGYWPSHRR 480
Consensus                   IETDYIFLVP TQAALYLHAA NAK---SGRT YSYLFSEPSR MPG---PYPS WMGADHADDL QYVFGKPFTT PLGYWPRHRD
```

		580		600		620		640	
BSAL - Human	VSKAMIAYWT	NFAKTGDPNM	GDSAVPTHWE	PYTTENSGYL	EITKKMGSSS	MKRSLRTNFL	RYWTLTYLAL	PTVTDQEATP	559
Bsal - Mouse	VSKAMIAYWT	NFARSGDPNM	GNSPVPTHWY	PYTLENGNYL	DITKTITSAS	MKEHLREKFL	KFWAVTFEVL	PTVTGDQDTL	559
bsal - Spotted gar	VSGYMIAYWT	NFAQTGDPNK	GESKVPAQWP	LFTSTGHKYL	EINNKINQNS	IKAMLRTRFI	HYWTSTYHSL	PPFS------	550
bsal.1 - European eel	VSRHMIAYWT	NFARTGDPNK	GESDVPVAWP	RFTGSEHQYL	EINEKMNRDS	VRQKLRTRFV	QFWSQTYALL	PTIK------	549
bsal.2 - European eel	VSRHMIAYWT	NFARTGDPNK	GESDVPVAWP	RFTGSEHQYL	EINEKMNRDS	VRQKLRTRFV	QFWTQTYALL	PTIK------	549
bsal.1 - Northern pike	LSEYMISYWT	NFARTGDPNQ	GVSKVPTTWP	EFSCSEHPYL	VINNKIKKSS	IKHNLRSQYV	KYWTSTYASL	PYVEVQ----	557
bsal.2 - Northern pike	VSKYFIAYWT	NFARTGDPNQ	GESKVPVTWP	AYTLSGQKYL	EINSKMNKNY	VHEKMRVRFV	NWWSNILPSL	PSE-------	592
bsal.1 - Atlantic cod	LSGYMIAYWT	NFAKTGDPSR	G-SKVPTIWP	PFTPSRHPYL	VINSDITKSS	VRFDLRAEYV	RYWTDTYNNM	PSVNSDQ---	555
bsal.2 - Atlantic cod	LSGYMIAYWT	NFAKTGDPNK	G-LKVPTTWP	KFTTSGHQFL	EINAKMNKDN	VRQKMRMRFV	HFWTSV---L	PSLQ------	551
bsal.3 - Atlantic cod	VSRYLIHYWT	NFAKSGDPNR	G-LDVPATWP	RFDSNSHQYL	EINSEMNRGY	VRQRLRMRYV	HFWGNI---L	PQL-------	551
bsal.4 - Atlantic cod	VSRYLIHYWT	NFAKSGDPNR	G-LDVPATWP	RFDSNSHQYL	EINSKMNPGY	VRQRLRMRYV	HFWGNI---L	PQL-------	551
bsal.5 - Atlantic cod	LSGYFISFWT	NFAWTGDPNV	GKSKVPLTWP	KFTCSEQKYL	EINSKMDRTS	VGQKMRSRFV	HFWTNTLPNL	PSVK------	552
bsal - Yellow catfish	VAKYMIAYWT	NFAKTGDPNI	GESEVPVAWP	KLTD-GHQFV	EINHDTGKNS	IKQKMRARFV	YFWSTTYTSF	PNV-------	547
bsal - Channel catfish	VSKYMIAYWT	NFAKTGDPNI	GESDVPVAWP	KLTE-GDQFV	EINNSMGKDS	VKQKMRARYV	YFWSTTYMSF	PNV-------	547
bsal.1 - Atlantic salmon	VSKYFIAYWT	NFARTGDPNK	GESNVPVTWP	AYTTSGQKYL	EINAKMNRNS	VHEKMRVRFV	NWWSN---TL	PSI-------	550
bsal.2 - Atlantic salmon	VSRYLIAYWT	NFARTGDPNN	GESKVPVTWP	AYTTSGQKYL	EINANMNKNY	VHEKMRVRFV	NWWSNIFPSL	PSV-------	555
bsal.1 - Rainbow trout	VSKYFIAYWT	NFARTGDPNK	GESNVPVTWP	AYTTSGQKYL	EINAKMNRNS	VHEKMRVRFV	NWWSN---TL	PSI-------	550
bsal.2 - Rainbow trout	VSRYLIAYWT	NFARTGDPNN	GESKVPVTWP	AYTTSGQKYL	EINAQMNKNY	VHEKMRVRFV	NWWSNIFPSL	PSV-------	555
bsal.1 - Largemouth bass	VSRYMIAYWT	NFAKTGDPNK	GE-KVPATWP	KFTSTGHQFL	EINSNMDKSY	VRQKMRMRYV	HFWTSV---L	PSL-------	572
bsal.2 - Largemouth bass	VSRYMIAYWT	NFAKTGDPNK	GE-KVPATWP	KFTSTGHQFL	EINSNMDKSY	VRQKMRMRYV	HFWTSV---L	PSL-------	572
bsal.3 - Largemouth bass	LSRYMIAYWT	NFARTGDPSK	GDNKVPVLWP	PFTKYHQPYL	TINHKITKSS	VSYDLRSNYV	AYWTKTYSLL	PSVKRNGGGE	555
bsal.1 - Asian seabass	LSGYMIAYWT	NFARTGDPSR	GNSKVPVPWP	PFTKNHRPYL	IINHKISKSS	ISYDLRSDYV	TYWTETYSSL	PTIKREEEDE	556
bsal.2 - Asian seabass	VSRYMIAYWT	NFAKTGDPNK	GDLSVPATWP	KFTNTGHQFL	EINSKMDKSY	ARQKMRMRYV	HFWASI---L	PSL-------	550
bsal.3 - Asian seabass	LSGYMIAYWT	NFARTGNPNK	GNLKVPVVWP	EFTSTGQQFL	DINAKMNESS	VGQEMRLRFL	HLWLNTLPSL	PSI-------	552
bsal.1 - Mandarin fish	VSRYMIAYWT	NFARTGDPNK	GEMNVPVTWP	RFTSTEHHFL	EINSKMDKSY	VRQKMRLRYV	HFWTDI---L	PNL-------	550
bsal.2 - Mandarin fish	VSRYMIAYWT	NFARTGDPNT	GNSRVPVLWP	PFTKLHQPYL	TINHNITKSS	VRYDLRSDYV	TYWTKTYSNL	PSVRREEEEE	554
bsal.1 - European seabass	LSGYLIAYWT	NFARTGDPNT	GNLKVPVTWP	EFTSIQHQFV	DLNAKMNESS	IGQEIRPQFV	RLWTSTLPSL	PSH-------	552
bsal.2 - European seabass	LSGYMIAYWT	NFAKTGDPSK	GDSRVPVPWP	AFTKYHHPYL	TINHKITKSS	VSYDLRSDYV	TYWTKTYSSL	PTIKREEEEE	561
bsal.1 - Pufferfish	LSGYMIAYWT	NFAKSGDPNS	GNSKVPALWP	PFTKYHHPYL	IINHKISKSS	IGYDLRSDHV	LYWRDTYSQL	PSVHQEEEVC	556
bsal.2 - Pufferfish	VSGYMIAYWT	NFAKTGDPNK	GDLSVPVTWP	QFTSTGHQYL	EIHSKMDSGY	VHQKMRMRYV	HFWTSV---L	PNL-------	549
bsal.3 - Pufferfish	VSRYMIAYWT	NFAKTGDPNK	GDLSVPVTWP	QFTSTGHQYL	EIHSKMDSGY	VHQKMRMRYV	HFWTSV---L	PNL-------	549
bsal.4 - Pufferfish	LSGNMIAYWT	NFARTGNPNK	GTLKVPVTWP	NFTSAGQQFL	DISAKMSESS	IGEKMRHNFV	RLWTKTLPGL	KS--------	551
bsal.1 - Japanese flounder	VSGYMIAYWT	NFARTGDPNK	GELSVPVTWP	EFTITGHKFL	DINSNMKNDS	VGQKMRARHV	HFWTSI---L	PSL-------	550
bsal.2 - Japanese flounder	VSGYMIAYWT	NFARTGDPNK	GELSVPVTWP	EFTITGHKFL	DINSNMKNDS	VGQKMRARHV	HFWTSI---L	PSL-------	458
bsal.3 - Japanese flounder	VSGYMIAYWT	NFARTGNPNK	GNLVVPVIWP	ECTSNGYQFL	DINSKMNESS	TGQEMRSRFV	RLWLSTLPGL	PSY-------	552
bsal.1 - Zebrafish	VSKYMIAYWS	NFAQTGDPNK	GESKVPVTWP	EFSNPGHQYL	DINNKMNKNN	VKQMLRTRLV	YYWTTVFASY	PTV-------	548
bsal.2 - Zebrafish	VSKYMIAYWT	NFAQTGNPNT	GESKVPVTWP	EFSNPGHQYL	DINNKINTNS	VKKMLRTRLV	YYWTTVFASY	PTV-------	549
bsal.1 - Medaka	LSQYMISYWT	NFAKTGDPNS	GNSRVPVPWP	PFTRNHHPYL	IINNNMSKSS	VSYDLRSYYV	NYWTQTYSSL	QKKTGKEFSQ	558
bsal.2 - Medaka	VSSYMIAYWT	NFAKTGDPNS	GDLKVPVAWP	KFQNTDHKFL	EINAKTNSES	VNEMLRLRYV	HFWASVFPKL	PS--------	551
bsal.3 - Medaka	VSEHMIAYWT	NFAKTGDPNS	GGLSVPVTWP	EFTNADHKFL	EINANINSAS	VREMLRLGYV	YFWTSYLPDL	PEVT------	558
bsal.1 - Common carp	VAKYMISYWT	NFAQTGDPNK	GESSVPVTWP	EFASNGHQYL	VINNKMNRDS	VKQMLRTRLV	YYWTNVFASF	PGVK------	551
bsal.2 - Common carp	VARYMISYWT	NFAQTGDPNK	GESSVPVTWP	EFASSGHQYI	DINNKMNRDS	VKQMLRTRLV	YYWTTVFASF	PAVQ------	551
bsal.1 - Tilapia nilotica	VSDYLISYWT	NFAKTGDPNN	GDSAVPATWP	TFTRSGEKFL	EINSDMNQNY	IRQEMRLRYV	HFWTSI---L	PSL-------	550
bsal.2 - Tilapia nilotica	VSGAMISYWT	NFAKTGDPNN	GGSSVPVNWP	KFTRSAPQFI	EINSDMNNNY	VQQKMRMPYV	NFWTRI---L	PSL-------	550
bsal.3 - Tilapia nilotica	VSGAMISYWT	NFAKTGDPNN	GGSSVPVNWP	TFTRSAPQFL	EIHSDMNNNY	VQQKMRMPYV	NFWTRI---L	PSL-------	550
Consensus	VSGYMIAYWT	NFAKTGDPNK	GESKVPVTWP	EFTSSGHQYL	EINSKMNKSS	VRQKMRMRYV	HFWTST--SL	PSV-------	

		660		680		700		720	
BSAL - Human	**VPPTGDSEAT**	**PVPPTGDSET**	**APVPPTGDSG**	**APPVPPTGDS**	**GAPPVPPTGD**	**SGAPPVPPTG**	**DSGAPPVPPT**	**GDSGAPPVPP**	639
Bsal - Mouse	**TPPEDDSEVA**	**PDPPSDDSQV**	**VPVPPT**----	----------	----------	----------	----------	----------	585
bsal - Spotted gar	----------	----------	----------	----------	----------	----------	----------	----------	550
bsal.1 - European eel	----------	----------	----------	----------	----------	----------	----------	----------	549
bsal.2 - European eel	----------	----------	----------	----------	----------	----------	----------	----------	549
bsal.1 - Northern pike	----------	----------	---**I**------	----------	----------	----------	----------	----------	558
bsal.2 - Northern pike	----------	----------	----------	----------	----------	----------	----------	----------	592
bsal.1 - Atlantic cod	----------	----------	----------	----------	----------	----------	----------	----------	555
bsal.2 - Atlantic cod	----------	----------	----------	----------	----------	----------	----------	----------	551
bsal.3 - Atlantic cod	----------	----------	----------	----------	----------	----------	----------	----------	551
bsal.4 - Atlantic cod	----------	----------	----------	----------	----------	----------	----------	----------	551
bsal.5 - Atlantic cod	----------	----------	----------	----------	----------	----------	----------	----------	552
bsal - Yellow catfish	----------	----------	----------	----------	----------	----------	----------	----------	547
bsal - Channel catfish	----------	----------	----------	----------	----------	----------	----------	----------	547
bsal.1 - Atlantic salmon	----------	----------	----------	----------	----------	----------	----------	----------	550
bsal.2 - Atlantic salmon	----------	----------	----------	----------	----------	----------	----------	----------	555
bsal.1 - Rainbow trout	----------	----------	----------	----------	----------	----------	----------	----------	550
bsal.2 - Rainbow trout	----------	----------	----------	----------	----------	----------	----------	----------	555
bsal.1 - Largemouth bass	----------	----------	----------	----------	----------	----------	----------	----------	572
bsal.2 - Largemouth bass	----------	----------	----------	----------	----------	----------	----------	----------	572
bsal.3 - Largemouth bass	**EE**----**DGAM**	**KREEEEEKLT**	**QLSA**------	----------	----------	----------	----------	----------	575
bsal.1 - Asian seabass	**KEK**-------	----------	----------	----------	----------	----------	----------	----------	559
bsal.2 - Asian seabass	----------	----------	----------	----------	----------	----------	----------	----------	550
bsal.3 - Asian seabass	----------	----------	----------	----------	----------	----------	----------	----------	552
bsal.1 - Mandarin fish	----------	----------	----------	----------	----------	----------	----------	----------	550
bsal.2 - Mandarin fish	**EVEEEEDGGM**	**KREG**---**KLI**	**QLSV**------	----------	----------	----------	----------	----------	575
bsal.1 - European seabass	----------	----------	----------	----------	----------	----------	----------	----------	552
bsal.2 - European seabass	----------	----------	----------	----------	----------	----------	----------	----------	561
bsal.1 - Pufferfish	**NLRQ**------	----------	--**GS**------	----------	----------	----------	----------	----------	562
bsal.2 - Pufferfish	----------	----------	----------	----------	----------	----------	----------	----------	549
bsal.3 - Pufferfish	----------	----------	----------	----------	----------	----------	----------	----------	549
bsal.4 - Pufferfish	----------	----------	----------	----------	----------	----------	----------	----------	551
bsal.1 - Japanese flounder	----------	----------	----------	----------	----------	----------	----------	----------	550
bsal.2 - Japanese flounder	----------	----------	----------	----------	----------	----------	----------	----------	458
bsal.3 - Japanese flounder	----------	----------	----------	----------	----------	----------	----------	----------	552
bsal.1 - Zebrafish	----------	----------	----------	----------	----------	----------	----------	----------	548
bsal.2 - Zebrafish	----------	----------	----------	----------	----------	----------	----------	----------	549
bsal.1 - Medaka	**R**---------	----------	--**SV**------	----------	----------	----------	----------	----------	561
bsal.2 - Medaka	----------	----------	----------	----------	----------	----------	----------	----------	551
bsal.3 - Medaka	----------	----------	----------	----------	----------	----------	----------	----------	558
bsal.1 - Common carp	----------	----------	----------	----------	----------	----------	----------	----------	551
bsal.2 - Common carp	----------	----------	----------	----------	----------	----------	----------	----------	551
bsal.1 - Tilapia nilotica	----------	----------	----------	----------	----------	----------	----------	----------	550
bsal.2 - Tilapia nilotica	----------	----------	----------	----------	----------	----------	----------	----------	550
bsal.3 - Tilapia nilotica	----------	----------	----------	----------	----------	----------	----------	----------	550
Consensus	----------	----------	----------	----------	----------	----------	----------	----------	

```
                                                    740                 760                 780                 800
                                                      |                   |                   |                   |
BSAL - Human                  TGDSGAPPVP PTGDSGAPPV PPTGDSGAPP VPPTGDAGPP PVPPTGDSGA PPVPPTGDSG APPVTPTGDS ETAPVPPTGD 719
Bsal - Mouse                  ---------- ---------- ---------- ---------- ---------- ---------- ---------- ---------- 585
bsal - Spotted gar            ---------- ---------- ---------- ---------- ---------- ---------- ---------- ---------- 550
bsal.1 - European eel         ---------- ---------- ---------- ---------- ---------- ---------- ---------- ---------- 549
bsal.2 - European eel         ---------- ---------- ---------- ---------- ---------- ---------- ---------- ---------- 549
bsal.1 - Northern pike        ---------- ---------- ---------- ---------- ---------- ---------- ---------- ---------- 558
bsal.2 - Northern pike        ---------- ---------- ---------- ---------- ---------- ---------- ---------- ---------- 592
bsal.1 - Atlantic cod         ---------- ---------- ---------- ---------- ---------- ---------- ---------- ---------- 555
bsal.2 - Atlantic cod         ---------- ---------- ---------- ---------- ---------- ---------- ---------- ---------- 551
bsal.3 - Atlantic cod         ---------- ---------- ---------- ---------- ---------- ---------- ---------- ---------- 551
bsal.4 - Atlantic cod         ---------- ---------- ---------- ---------- ---------- ---------- ---------- ---------- 551
bsal.5 - Atlantic cod         ---------- ---------- ---------- ---------- ---------- ---------- ---------- ---------- 552
bsal - Yellow catfish         ---------- ---------- ---------- ---------- ---------- ---------- ---------- ---------- 547
bsal - Channel catfish        ---------- ---------- ---------- ---------- ---------- ---------- ---------- ---------- 547
bsal.1 - Atlantic salmon      ---------- ---------- ---------- ---------- ---------- ---------- ---------- ---------- 550
bsal.2 - Atlantic salmon      ---------- ---------- ---------- ---------- ---------- ---------- ---------- ---------- 555
bsal.1 - Rainbow trout        ---------- ---------- ---------- ---------- ---------- ---------- ---------- ---------- 550
bsal.2 - Rainbow trout        ---------- ---------- ---------- ---------- ---------- ---------- ---------- ---------- 555
bsal.1 - Largemouth bass      ---------- ---------- ---------- ---------- ---------- ---------- ---------- ---------- 572
bsal.2 - Largemouth bass      ---------- ---------- ---------- ---------- ---------- ---------- ---------- ---------- 572
bsal.3 - Largemouth bass      ---------- ---------- ---------- ---------- ---------- ---------- ---------- ---------- 575
bsal.1 - Asian seabass        ---------- ---------- ---------- ---------- ---------- ---------- ---------- ---------- 559
bsal.2 - Asian seabass        ---------- ---------- ---------- ---------- ---------- ---------- ---------- ---------- 550
bsal.3 - Asian seabass        ---------- ---------- ---------- ---------- ---------- ---------- ---------- ---------- 552
bsal.1 - Mandarin fish        ---------- ---------- ---------- ---------- ---------- ---------- ---------- ---------- 550
bsal.2 - Mandarin fish        ---------- ---------- ---------- ---------- ---------- ---------- ---------- ---------- 575
bsal.1 - European seabass     ---------- ---------- ---------- ---------- ---------- ---------- ---------- ---------- 552
bsal.2 - European seabass     ---------- ---------- ---------- ---------- ---------- ---------- ---------- ---------- 561
bsal.1 - Pufferfish           ---------- ---------- ---------- ---------- ---------- ---------- ---------- ---------- 562
bsal.2 - Pufferfish           ---------- ---------- ---------- ---------- ---------- ---------- ---------- ---------- 549
bsal.3 - Pufferfish           ---------- ---------- ---------- ---------- ---------- ---------- ---------- ---------- 549
bsal.4 - Pufferfish           ---------- ---------- ---------- ---------- ---------- ---------- ---------- ---------- 551
bsal.1 - Japanese flounder    ---------- ---------- ---------- ---------- ---------- ---------- ---------- ---------- 550
bsal.2 - Japanese flounder    ---------- ---------- ---------- ---------- ---------- ---------- ---------- ---------- 458
bsal.3 - Japanese flounder    ---------- ---------- ---------- ---------- ---------- ---------- ---------- ---------- 552
bsal.1 - Zebrafish            ---------- ---------- ---------- ---------- ---------- ---------- ---------- ---------- 548
bsal.2 - Zebrafish            ---------- ---------- ---------- ---------- ---------- ---------- ---------- ---------- 549
bsal.1 - Medaka               ---------- ---------- ---------- ---------- ---------- ---------- ---------- ---------- 561
bsal.2 - Medaka               ---------- ---------- ---------- ---------- ---------- ---------- ---------- ---------- 551
bsal.3 - Medaka               ---------- ---------- ---------- ---------- ---------- ---------- ---------- ---------- 558
bsal.1 - Common carp          ---------- ---------- ---------- ---------- ---------- ---------- ---------- ---------- 551
bsal.2 - Common carp          ---------- ---------- ---------- ---------- ---------- ---------- ---------- ---------- 551
bsal.1 - Tilapia nilotica     ---------- ---------- ---------- ---------- ---------- ---------- ---------- ---------- 550
bsal.2 - Tilapia nilotica     ---------- ---------- ---------- ---------- ---------- ---------- ---------- ---------- 550
bsal.3 - Tilapia nilotica     ---------- ---------- ---------- ---------- ---------- ---------- ---------- ---------- 550

Consensus                     ---------- ---------- ---------- ---------- ---------- ---------- ---------- ----------
```

		820			
BSAL - Human	SGAPPVPPTG	DSEAAPVPPT	DDSKEAQMPA	VIRF	753
Bsal - Mouse	----------	----------	DDSVEAQMPA	TIGF	599
bsal - Spotted gar	----------	----------	--------NS	TAEY	556
bsal.1 - European eel	----------	----------	--------SP	S-AE	554
bsal.2 - European eel	----------	----------	--------SP	S-AE	554
bsal.1 - Northern pike	----------	----------	----------	----	558
bsal.2 - Northern pike	----------	----------	----------	----	592
bsal.1 - Atlantic cod	----------	----------	----------	----	555
bsal.2 - Atlantic cod	----------	----------	--------SV	KMYE	557
bsal.3 - Atlantic cod	----------	----------	--------SL	LKFL	557
bsal.4 - Atlantic cod	----------	----------	--------SL	LKFL	557
bsal.5 - Atlantic cod	----------	----------	----------	---Y	553
bsal - Yellow catfish	----------	----------	----------	---N	548
bsal - Channel catfish	----------	----------	----------	---N	548
bsal.1 - Atlantic salmon	----------	----------	----------	----	550
bsal.2 - Atlantic salmon	----------	----------	----------	----	555
bsal.1 - Rainbow trout	----------	----------	----------	----	550
bsal.2 - Rainbow trout	----------	----------	----------	----	555
bsal.1 - Largemouth bass	----------	----------	--------PV	IISE	578
bsal.2 - Largemouth bass	----------	----------	--------PV	IISE	578
bsal.3 - Largemouth bass	----------	----------	----------	----	575
bsal.1 - Asian seabass	----------	----------	----------	----	559
bsal.2 - Asian seabass	----------	----------	--------PQ	NIYE	556
bsal.3 - Asian seabass	----------	----------	--------Q-	----	553
bsal.1 - Mandarin fish	----------	----------	--------PI	NNSE	556
bsal.2 - Mandarin fish	----------	----------	----------	----	575
bsal.1 - European seabass	----------	----------	--------AV	ADAQ	558
bsal.2 - European seabass	----------	----------	----------	----	561
bsal.1 - Pufferfish	----------	----------	----------	----	562
bsal.2 - Pufferfish	----------	----------	--------NV	TISE	555
bsal.3 - Pufferfish	----------	----------	--------NV	TISE	555
bsal.4 - Pufferfish	----------	----------	--------HI	ALDN	557
bsal.1 - Japanese flounder	----------	----------	--------P-	----	551
bsal.2 - Japanese flounder	----------	----------	--------P-	----	459
bsal.3 - Japanese flounder	----------	----------	--------HH	TDAL	558
bsal.1 - Zebrafish	----------	----------	--------R-	---N	550
bsal.2 - Zebrafish	----------	----------	--------R-	---N	551
bsal.1 - Medaka	----------	----------	----------	----	561
bsal.2 - Medaka	----------	----------	----------	---Q	552
bsal.3 - Medaka	----------	----------	----------	--SR	560
bsal.1 - Common carp	----------	----------	--------K-	---N	553
bsal.2 - Common carp	----------	----------	--------K-	---S	553
bsal.1 - Tilapia nilotica	----------	----------	--------PT	VTSE	556
bsal.2 - Tilapia nilotica	----------	----------	--------PT	VVSE	556
bsal.3 - Tilapia nilotica	----------	----------	--------PT	VVSE	556
Consensus	----------	----------	----------	----	
