# Supplementary material for: Comparative Study of the Molecular Characterization, Evolution, and Structure Modeling of Digestive Lipase Genes Reveals the Different Evolutionary Selection Between Mammals and Fishes
Source: Front Genet. 2022 Aug 4;13:909091. doi: 10.3389/fgene.2022.909091 (PMC9386070; doi:10.3389/fgene.2022.909091)
Supplement: Supplementary file 4 [file Datasheet1.PDF]

Atlantic salmon (*Salmo salar*)

exon 1：
ATGTCTGTCGTTAATTGTACTGGTTACTTATTCATTTTTTTGACAAGGGTGACGAGAACTGGCTTATTGACATGTGCAAA

exon 2：
ACCATACTGACAGAGGAAGAGGTCAACTGCATCTGTGTGGACTGGAAGAAGGGGGGGCTGACCCTCTGCACACAGGCAGCCAATAACATCTGAGTC
GTTGGTGCCCAGGTGGCCCACATGATTGACGTCTTCTGG

exon 3：
GACGTCTATTGGCAGAAGGCTAGCATGGCCCACGTTATCGGACAATCTGGGAGCAGACGCTGCTGGGGAGGTGGGCCGCAGGATCAGCAGGCTAGGA
CGCATCACAG

exon 4：
GGCTGGACCCAGCACAGCCCTGCTTCCAGGGCCTCATTGCAACGGTGCGCCTGGACCCCTCTGATGCCACGTTTTGTAGATGTCATTCACACTGACAC
GCTGCCCTTCATCCCTTATGTTG

exon 5：
GCAAGGGCATATCTCAGGCTGTTGGCCACATTGACCTCTACCCTAACGGAGGAGAGCACATGCCTGGCTGTGACAAGAACATTGTCTCTACCATTGTG
GACATTGACTGCCTTTGGGAAGGGGAGAACTGATGCATATTTGTACTGTGTCTTGAAATCTGTAGAATTGGGGGAAAATGCTTAAAAGGGGAAGATAG
ATGAGGATCAACCGCAAGGGAAAATAACTCCCGATTGCGTGATAGAAATGTAAATGTAACTTCCGATTTGCGCTGACATGCAGCAGCGGCAATGTGAA
TGCAGTCTCCTCTAATGCCGGAACATTGCCTTTAAATTTCAATAACACTAATGCTGAACTTCTGCGTTACGGATTGAATAGAGCCAGGGGAGAACGACT
GCCCCCTCTCATTGAAGCTACAGAAGTTGAATGCCAACTGCAGTACTGCAGGCATTGTTAGCGGAAAACAGGATCCCCCCATTACAGCGAATGGGAAA
AATGACAATCTTCTTAAAAAAACGAAATAGAATGATACAAATAATTGCTTCTAATAAACCAGATGTATGTCCTTGAACTGATAACTTCAAAATTGCACAC
AGAATAAGTTATAAGGATAATTGAGTTGCTAATGGCAACCTGCAGTACCCCAGGTTGGCCTTCAATTTGAGCTACTCAATGAGAGGTGGCAGCACTGA
GTTAGCCAGCAACATCACTTCATGGATTAGCTCAAACTTCACGTTATTCCTCCATGTGACACTATCTTAAGACTTAATTTGGCTTGAATGCGCTATTGAG
TCTTCACATAGGAATGAATGGTGTCACTTGATCGATGTATGAATGGCCAAAGCCAGTCAGTGAATAGAGACCCCGATCTATTAACCAGCCGTTACAAGT
TGATGTAATGTAACTTCCTATGTAGTGTAAGATTACCCACCAAGGTCAGAACTAGCATCATAGGCACTCCATGTCACAATGACACACACATTTTCTTGTC
TGGCCATCTTAAAATGTATCCCCTGGATTATTGGAGTGGTACCAATGGTAAAACCATAA

rainbow trout (*Oncorhynchus mykiss*)

exon 1：
ATGATTGACGTCTTCTGG

exon 2：
GATGTCTACTGGCCGAAGGCTAGCATGGTCCACGTTATCGGACAGTCTGGGAGCACACACTGCTGGGGAGGTGGGCCGCAGGATCAGCAGGCA
AGGACGCGTCACAG

exon 3：
GGCTGGACCCAGCACAGCCCTGCTTCCAGGGCCTCATTGCCACGGTACGCCTGGACGCCTCCAATGCCACATTTGTAGATGTCATTCACACTGACAC
GCTGCCCTTCATCTCTTATGTTG

exon 4：
ACAAGGGCATATCGCAGGCTGTTGGCCATATTGACTTCTACCCTAACGGAGGAGAGCACATGCCTGGCTGTGACAAGAACATTGTCTCTACCATTGTG
GACATTGACTGCCTTTGGGAAGGGGAGAACTGACGCATATTTGTACTGT

exon 5：
ACACGCGCAATGTTGTTGCCTGCAACCATCTCTGATCCTTCAAGTACTACAATGACAGACTCGTAAACCCTGAAGGCTTTACGGGATACAGGTTTTAA

Nile tilapia (*Oreochromis niloticus*)

exon 1：
ATGGCCACTACTGACCAAGTGCTTAGTGAATACCTCAGTAGAAACCCCAAAAAGAAAAGGAACAAGGAAAGGAAATATCGTGAAAGGACAGGCCCC
TACACG

exon 2：
GTTCAGGGGCAAAATATCCCTGTTGGCCCTTCCCGCTCTGTAGATGTATTCTAAGGAATTTCCTTTGCTGCCAAACCTGGACCTTTCGAAAAACCCAAA
CCTCACCCTGGCTGGGATG

exon 3：
GCATACTGAAGGCAACTAAGTTTGCTAGGAGATGCCTCCAAATCAACATGATCGACCCTGCATCAAGTCTTGGTAGTGAAGACTCCCTCTACCTCAAC
ATCTGGGTTCCTCACAGCGAAAATG

exon 4：
TCTCATCAAACCTCCCAGTGATGATTTGATTTTATGGAGGGGGCTTCATGTCCGGTGGTTCCAAGGGGGTAAACTTTCTCAGGAAATTGCAGACAGGG
GCGATGTCATCATAGTGTCTGTGGGATACCGTGTGGGAACCTTGGGATTCCTCAGTACAGGAGACTCTAGCTTTCCTG

exon 5：
GAAACTATGGTCTGTGGGACCAGCATGCTGCCATCGCCTGGGTACGCAGGAATATCCAGTCATTTGGAGGTGACCCTGACAACATCACCCTCTTTGGG
GAGTCTGCAGGTGGAGCTAGTGTTTGCTTCCAG

exon 6：
ACGCTCACTCCATATAACAAAGGGTTGATCAGGAAAGCTATCTCCCAGAGCGGTGTCGGTCTTTGTCAATGGGCTTATAACAAAAATCCACATAAGATT
GCAGTGGAG

exon 7：
GTTGCTGAGAAAGTGGGCTGCCCAACTGATGAAAGTATGGTGGCCTGTTTAAAATCAACTAATGCTGTGACTCTTACCATGGCTGCTCCCCCCCCAT

exon 8：
ATCCTGGAGTTCTCAGCCAGTTGATGTCTTCTGTTGTTGATGGAGACTTCCTGCCTGATGAGCCTGGCAACTTGTTCCACAACGCTGCTGAAATAGACT
ACCTTGCAGGTGTTAATGACATGGATGGCCGATCCTTCACTGCACAAGACATTCCTTCCCTTTCTGACAAGAATAAAGAGATTCCAGTGTAAG

exon 9：
ACGTAAAAAGACTCCTTGCTGCTTATACTAAAAAGAAAGGGGAAGCTGGTTTGGATATTGCCTTTGCTGAATACATATCCAACTGGGATTCAACACCCA
GTCAGGACACAATCAAGAGAACTGCTGTGGAAATTGGGACAGATTACATCTTCCTGGTTCCAACAGAGGCTGCAGTTTATCTACATGCTGCTAACGCC
AA

exon 10：
GTCTGGTCACACTTATTTCTGCGTAATGTCTGAGCCCAGATTATTGGCTGGACCAAACAAACCATACCATGACTGGGTGGGAGCAGACCACACTGATG
ACCCTCAGTATGCGTTTGGGAAACTCTTCACCACACCAAGTGCTTATGGGCAGAGACACAGAGACCTGTCTGGCTACATGATTGCCTACTGGACTAAC
TTTGCTAGAACTGG

exon 11：
AGACCCCAATAAAGAACACCTGACTGTTCCTGTGACCTGGCCTAAGTTTACCACCAGTGGACAAAACTACCTGGACATCAATGCAAAGATGAATGAA
AGCTCTGTTGGAAAGCAAATGAGGCTTTGCTTTGTTCGCCTTTGGACAACCACCCTTCCCAGCCTCCCATCCCATGGTGCTACAGATGTTTAATTATGC
ATTTTTAATTTAAATAATACATCATATTCGATTATATAATACAAAAAAGTGGGCAAAAATGTGTTTGGCTT

Supplementary Fig. 1. The pseudogenes of *pl* genes in Atlantic salmon and rainbow trout, the pseudogene of *bsal* gene in Nile tilapia. The premature termination codons were marked with red. The termination codon (TGA) was present in the second exon in Atlantic salmon. The termination codon (TGA) was present in the third exon in rainbow trout. The termination codon (TAA) was present in the second exon in Nile tilapia.
